# Exploring Emergent Properties in Enzymatic Reaction Networks: Design and Control of Dynamic Functional Systems

**DOI:** 10.1021/acs.chemrev.3c00681

**Published:** 2024-03-04

**Authors:** Souvik Ghosh, Mathieu G. Baltussen, Nikita M. Ivanov, Rianne Haije, Miglė Jakštaitė, Tao Zhou, Wilhelm T. S. Huck

**Affiliations:** Institute for Molecules and Materials, Radboud University, Heyendaalseweg 135, 6525 AJ Nijmegen, The Netherlands

## Abstract

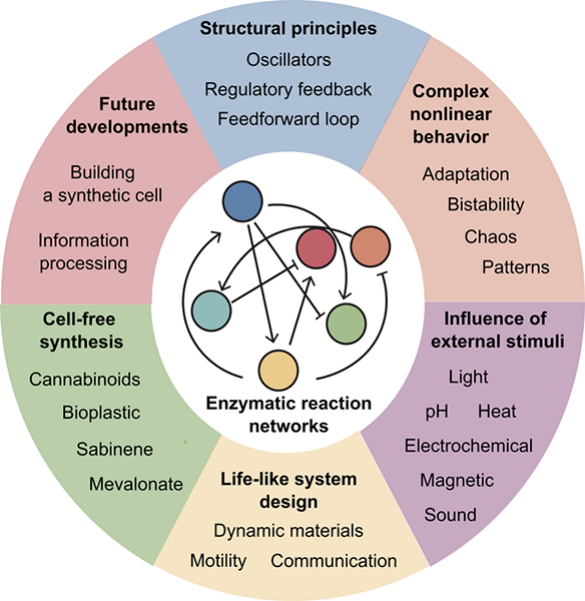

The intricate and complex features of enzymatic reaction networks (ERNs) play a key role in the emergence and sustenance of life. Constructing such networks *in vitro* enables stepwise build up in complexity and introduces the opportunity to control enzymatic activity using physicochemical stimuli. Rational design and modulation of network motifs enable the engineering of artificial systems with emergent functionalities. Such functional systems are useful for a variety of reasons such as creating new-to-nature dynamic materials, producing value-added chemicals, constructing metabolic modules for synthetic cells, and even enabling molecular computation. In this review, we offer insights into the chemical characteristics of ERNs while also delving into their potential applications and associated challenges.

## 1 Introduction

All key functions of living systems, such as metabolism, reproduction, sensing the environment, adaptation, and homeostasis, are enabled by enzymatic reaction networks (ERNs). In metabolic networks, the activities of many enzymatic reactions are finely tuned to provide responsiveness and controlled behavior. Highly complex emergent properties such as chemotaxis,^1,2^ cell division, and the cell cycle^2^ are all orchestrated by enzymatic networks. Signaling pathways consisting of cascades of kinases and phosphatases interlock to form complex networks that allow cells to process information from the environment and activate suitable genetic programs.^3−5^

In the field of synthetic biology, enormous progress has been made in appropriating and harnessing some of the enzymatic networks in living cells to create, for example, artificial proteolysis signaling pathways and control intracellular self-assembly.^6−8^ Progress in synthetic biology has been reviewed earlier.^9,10^ Here, we wish to focus on synthetic enzymatic reaction networks outside a cellular context. These *in vitro* systems offer the key advantage of precise control over all components. This control enables deep and detailed chemical understanding of the dynamics of ERNs, which is a prerequisite for designing systems with emergent properties. The bottom-up construction of functional ERNs could then be used to create life-like materials, where the emergent dynamics of the networks can be coupled to material properties. Ultimately, ever more complex artificial ERNs may be used in the construction of synthetic cells, composed of multiple networks at the genetic and metabolic level, all compartmentalized within a synthetic compartment.

**Figure 1:**
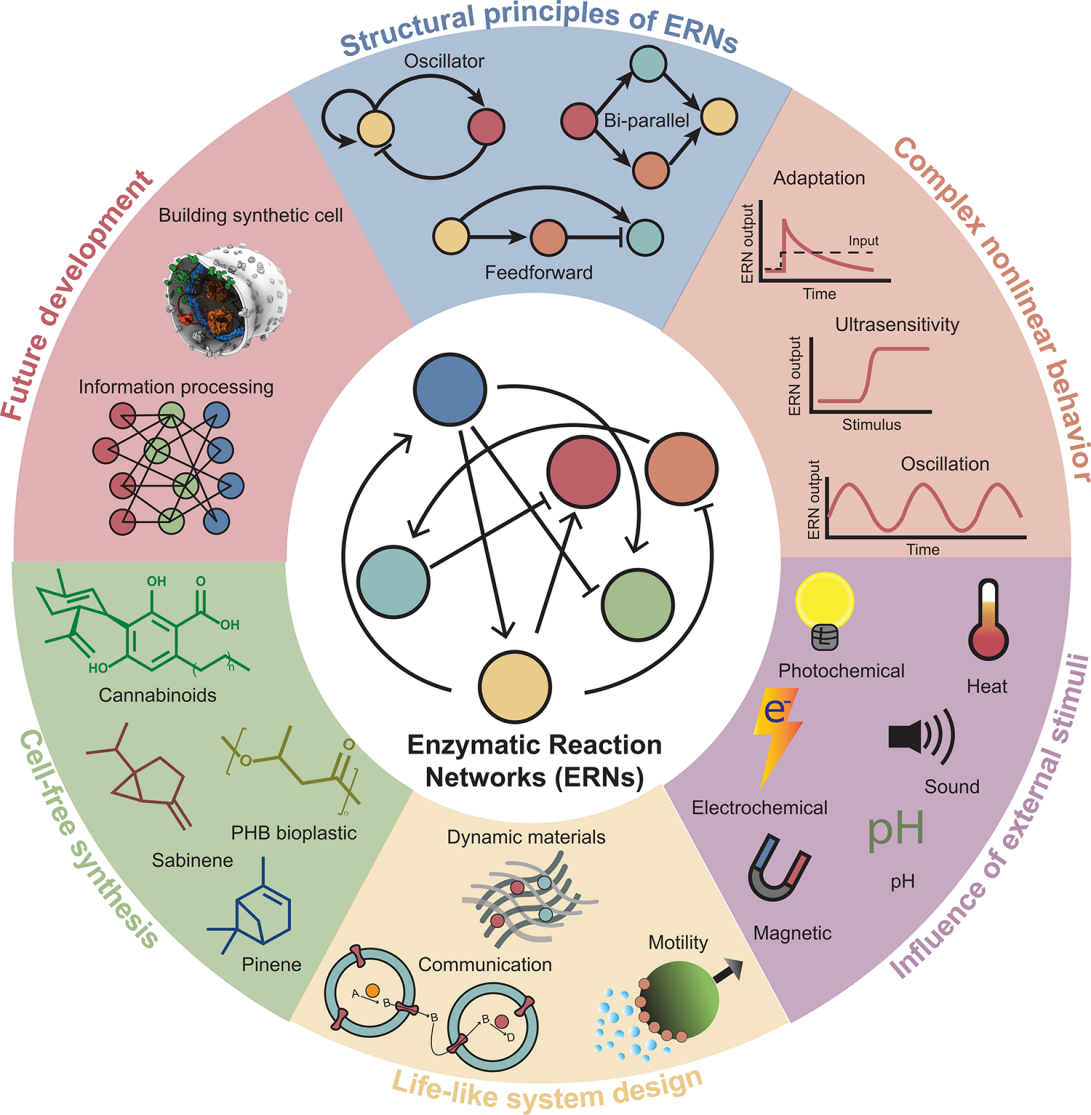
Overview of the topics discussed in the review. The image in “building synthetic cell” shows a cartoon representation of a synthetic cell (Credit: Graham Johnson/BaSyC consortium).

To achieve these long-term ambitions, the field has started to develop robust methods to construct ERNs and control their temporal and spatial dynamics. In this review, we will provide an overview of the progress to date, discuss challenges ahead (Figure 1), and finish with an overview table listing all enzymes used in enzymatic reaction networks (Table 1). The structure of our review is as follows: we start in section 2 with an overview of the structure of networks by discussing topology, motifs, and kinetics, highlighting approaches to modeling the dynamic properties of ERNs as well as opportunities for computational design of functional ERNs. In section 3, we provide examples of artificial ERNs with nonlinear dynamic behavior. In section 4, we discuss various means to control enzymatic activity via external stimuli such as pH, light, magnetic fields, and sound. Combined, these design and control principles have led to progress in enzymatically powered dynamic materials with life-like properties, which are reviewed in section 5. Having established the key aspects of designing and controlling ERNs, we devote a final set of sections on potential areas of application. In section 6, we review how ERNs can be used in the cell-free synthesis of valuable compounds. In section 7, we summarize the ambitious efforts to build a synthetic cell from the bottom up and highlight new developments where the information processing typically reserved for living systems is harnessed in synthetic ERNs. We conclude the review with a brief summary of the key goals and potential bottlenecks for future research.

**Table 1:** Overview of Enzymes Mentioned in the Review

enzymes	Enzyme Commission number	class	mode of action	section	ref
alcohol dehydrogenase (ADH)	1.1.1.1	oxidoreductase	ADH converts primary alcohols to aldehydes	5.2	(165)
l-lactate dehydrogenase (LDH)	1.1.1.27	oxidoreductase	LDH converts pyruvate to lactate and vice versa in glycolysis	5.2	(164)
l-malate NADP^+^ oxidoreductase (ME)	1.1.1.40	oxidoreductase	ME catalyzes oxidative decarboxylation of l-malate to form pyruvate (reversible)	4.3	(105)
hydroxymethylglutaryl-CoA reductase (HMGR)	1.1.1.88	oxidoreductase	HMGR converts 3-hydroxy-3-methylglutaryl-CoA (HMG-CoA) to mevalonic acid	6.1	(208)
glucose-6-phosphate dehydrogenase (G6PDH)	1.1.1.49	oxidoreductase	G6PDH converts glucose-6-phosphate to 6-phosphogluconolactone in pentose-phosphate pathway	5.2, 5.3, and 6.1	(164), (179), (202)
hydroxybutyryl-CoA dehydrogenase (Hbd)	1.1.1.157	oxidoreductase	Hbd catalyzes conversion of acetoacetyl-CoA to hydroxybutyryl-CoA and vice versa	6.1	(205)
l-lactate oxidase (LO)	1.1.3.2	oxidoreductase	LO is a FMN-containing enzyme that catalyzes conversion of lactate to pyruvate	5.2	(164)
glucose oxidase (GOx)	1.1.3.4	oxidoreductase	GOx catalyzes oxidation of d-glucose to d-gluconic acid by oxygen	3.1, 3.3, 4.2, 4.5, and 5.1	(60), (61), (78), (96), (99), (110), (139), (147), (185)
choline oxidase (COx)	1.1.3.17	oxidoreductase	COx is a flavoprotein, which catalyzes formation of betaine from choline	5.1	(147)
glyceraldehyde-3-phosphate dehydrogenase (Gap)	1.2.1.12	oxidoreductase	Gap converts glyceraldehyde 3-phosphate to 1,3-bisphosphoglycerate during glycolysis	6.1	(200)
pyruvate dehydrogenase (PDH)	1.2.4.1	oxidoreductase	PDH catalyzes the reaction of pyruvate and a lipoamide to give dihydrolipoamide and CO_2_ and is involved many metabolic pathways like glycolysis and TCA cycle	6.1	(200), (201), (203)
pyruvate:ferredoxin oxidoreductase (PFOR)	1.2.7.1	oxidoreductase	PFOR converts acetyl-CoA to pyruvate in many metabolic cycles including pyruvate metabolism, propanoate metabolism, and butanoate metabolism	6.2	(219)
d-amino acid oxidase (DAAO)	1.4.3.3	oxidoreductase	DAAO converts d-amino acids to 2-oxo carboxylates and is involved in d-amino acid metabolism	4.2	(95)
sarcosine oxidase (SOx)	1.5.3.1	oxidoreductase	SOx catalyzes formation of glycine from sarcosine by oxidative demethylation	5.1 and 5.2	(147), (165)
NADH oxidase (NoxE)	1.6.3.4	oxidoreductase	water-forming NoxE is a flavoprotein that specifically oxidizes NADH, not NADP	6.1	(199−202)
urate oxidase (UOx)	1.7.3.3	oxidoreductase	UOx is involved in the allantoin pathway and converts uric acid to 5-hydroxyisourate	5.1	(147)
catalase (Cat)	1.11.1.6	oxidoreductase	Cat is a peroxidase and is involved in biosynthesis of tryptophan and secondary metabolites	5.2	(161−163), (165), (168−170), (184), (185), (187)
horseradish peroxidase (HRP)	1.11.1.7	oxidoreductase	catalyzes oxidation of various organic substrates by hydrogen peroxide; heme containing glycoprotein with many isoforms	3.3, 4.2, 4.5, 5.1, and 5.3	(74−76), (97), (110), (111), (140), (178), (183), (184)
NiFe hydrogenase	1.12.2.1	oxidoreductase	NiFe hydrogenase oxidizes H_2_ to H^+^ (reversible), present in prokaryotes	3.3 and 4.3	(79), (106)
renillaluciferase (RLuc)	1.13.12.5	oxidoreductase	RLuc converts coelenterazine to excited coelenteramide and emits blue light	5.3	(176)
ferredoxin NADP^+^ reductase (FNR)	1.18.1.2	oxidoreductase	FNR is a flavoprotein involved in photosynthesis	4.3	(105)
Mo-dependent nitrogenase	1.18.6.1	oxidoreductase	Mo-dependent nitrogenase involved in nitrogen fixation, catalyzes ammonia formation from nitrogen	4.3	(106)
acetyl-CoA acetyltransferase (ACAT)	2.3.1.9	transferase	ACAT is present in many metabolic pathways, where it catalyzes formation of acetoacetyl-CoA from acetyl-CoA	6.1	(208)
PHB synthase (PhaC)	2.3.1.304	transferase	Phac catalyzed formation of Bioplastic PHB from 3-hydroxybutyryl-CoA	6.1	(202)
hydroxymethylglutaryl-CoA synthase (HMGS)	2.3.3.10	transferase	HMGS catalyzes formation of HMG-CoA from acetyl-CoA and acetoacetyl-CoA in the mevalonate pathway	6.1	(208)
glycogen phosphorylase b (GPb)	2.4.1.1	transferase	GPb breaks down glycogen to form glucose-1-phosphate and is involved in starch and sucrose metabolism	5.3	(179)
geranyl diphosphate synthase (GPPS)	2.5.1.1	transferase	GPPS forms geranyl diphosphate from the condensation of dimethylallyl diphosphate (DMAPP) and isopentenyl diphosphate (IPP)	6.1	(204), (208)
hexokinase (HK)	2.7.1.1	transferase	hexokinase phosphorylate d-hexose sugars in the presence of ATP, thus playing a very important role in glycolysis	4.3 and 5.2	(104), (163)
mevalonate kinase (MK)	2.7.1.36	transferase	MK phosphorylates mevalonate to mevalonate-6-phosphate	6.1	(208)
protein kinase A (PKA)	2.7.1.37	transferase	PKA initiates phosphorylation of serine residues present in the peptide chain	5.1	(145)
pyruvate kinase (PK)	2.7.1.40	transferase	PK catalyzes the last step of glycolysis by transferring the phosphate group from PEP to ADP	5.2	(163)
phosphomevalonate kinase (PMK)	2.7.4.2	transferase	PMK catalyzes the phosphorylation of mevalonate-6-phosphate to form diphosphomevalonate in the mevalonate pathway	6.1	(208)
esterase (Est, PLE for pig liver esterase)	3.1.1.1	hydrolase	Est catalyzes hydrolysis of the ester bonds of carboxyl esters	3.1, 4.1, and 4.5	(59), (60), (65), (93), (110)
phospholipase A2 (PLA2)	3.1.1.4	hydrolase	PLA2 catalyzes cleavage of phospholipids	5.3	(176)
acetylcholine esterase (AchE)	3.1.1.7	hydrolase	AchE breaks down the ester bond of neurotransmitter acetylcholine to form choline and acetic acid	4.2 and 5.1	(99), (147)
phosphatase	3.1.3.1/3.1.3.2	hydrolase	phosphatases dephosphorylate phosphate esters	5.1	(139), (141)
amyloglucosidase (AMG)	3.2.1.3	hydrolase	AMG breaks down starch to glucose, hence involved in starch metabolism	4.5	(110)
lactase	3.2.1.108	hydrolase	lactase catalyzes the conversion of lactose to galactose and glucose	5.3	(183)
aminopeptidase (Ap)	3.4.11.2	hydrolase	catalyzes cleavage of N-terminal amino acids from peptides and is involved in glutathione metabolism	3.2	(67)
chymotrypsin (Cr)	3.4.21.1	hydrolase	Cr is a serine protease that cleaves peptides on the C terminal of phenylalanine, tyrosine, tryptophan, and leucine amino acids	3.2 and 5.1	(70), (73), (146)
trypsin (Tr)	3.4.21.4	hydrolase	Tr is a serine protease that cleaves peptides on the C terminal of arginine and lysine amino acids	3.2, 4.1, 4.4, and 5.2	(42), (67−73), (86), (89), (146)
elastase (Els)	3.4.21.36	hydrolase	elastase breaks down elastin (responsible for the elasticity of connective tissue) and cleaves after glycine, alanine, and valine amino acids	3.2	(73)
proteinase K	3.4.21.64	hydrolase	proteinase K is a serine protease with a broad spectrum of cleavage site preferences	5.3	(175)
matrix metalloproteinase 2 (MMP 2)	3.4.24.24	hydrolase	MMP 2 is an endopeptidase and cleaves collagens type IV, V, VII, and X; also known as gelatinase A	5.1	(145)
matrix metalloproteinase 9 (MMP 9)	3.4.24.35	hydrolase	similar to MMP 2; also known as gelatinase B	5.1	(17)
urease (Ur)	3.5.1.5	hydrolase	catalyzes the hydrolysis of urea to carbon dioxide and ammonia, which basifies the solution	3.1, 4.1, 4.2, 5.1, and 5.2	(51−54), (57−59), (61), (93), (97−99), (110), (157−160), (171), (185), (187), (189)
apyrase	3.6.1.5	hydrolase	apyrase hydrolyses di- and triphosphate nucleotides to monophosphate nucleotides	5.3	(179)
oxaloacetate acetylhydrolase (OAH)	3.7.1.1	hydrolase	OAH breaks down oxaloacetate to form oxalate and acetate	6.2	(219)
pyrophosphomevalonate decarboxylase (PMD)	4.1.1.33	lyase	PMD catalyzes the last step of the mevalonate pathway, where it converts diphosphomevalonate to isopentenyl diphosphate	6.1	(208)
carbonic anhydrase (CA)	4.2.1.1	lyase	CA catalyzes the equilibrium between carbon dioxide and carbonic acid	4.3	(105)
fumarase (FumC)	4.2.1.2	lyase	FumC converts malate to fumarate (reversible) and is involved in the TCA cycle and pyruvate metabolism	4.3	(105)
limonene synthase (LS)	4.2.3.16	lyase	limonene synthase catalyzes limonene formation from geranyl diphosphate	6.1	(200), (208)
pinene synthase	4.2.3.121	lyase	pinene synthase catalyzes the conversion of geranyl diphosphate to pinene	6.1	(200)
l-aspartate ammonia-lyase (AspA)	4.3.1.1	lyase	AspA converts l-aspartate to fumarate (reversible) and is involved in amino acid metabolism	4.3	(105)
isopentenyl pyrophosphate isomerase (IDI)	5.3.3.2	isomerase	IDI catalyzes the conversion of IPP to DMAPP	6.1	(208)
phosphoglucomutase (PMG)	5.4.2.2	isomerase	PMG catalyzes the isomerization of glucose-1-phosphate to glucose-6-phosphate and is involved in many metabolic pathways including glycolysis and the pentose phosphate pathway	5.3	(179)
acetate-CoA ligase (ACS)	6.2.1.1	ligase	ACS catalyzes acetyl-CoA formation from acetate and CoA in the presence of ATP	6.2	(219)
pyruvate carboxylase (PYC)	6.4.1.1	ligase	PYC catalyzes the carboxylation of pyruvate to form oxaloacetate in the TCA cycle	6.2	(219)
cytochrome *C* oxidase	7.1.1.9	translocase	cytochrome *C* oxidase catalyzes the translocation of hydrons and is involved in oxidative phosphorylation pathways	4.2	(95)
F-type ATP synthase	7.1.2.2	translocase	F-type ATP synthase forms ATP from ADP and phosphate (Pi)	7.1	(227), (228)

## 2 Structural Principles of Enzymatic Networks

Despite a huge variety in enzymes and enzymatic reactions, many of the enzymatic reaction networks (ERNs) found in living cells feature recurring topologies and motifs. By focusing on these patterns, it becomes clear how certain functionalities arise from specific groupings of enzymes, and similar or alternative networks can be identified.

### 2.1 Network Topology

**Figure 2:**
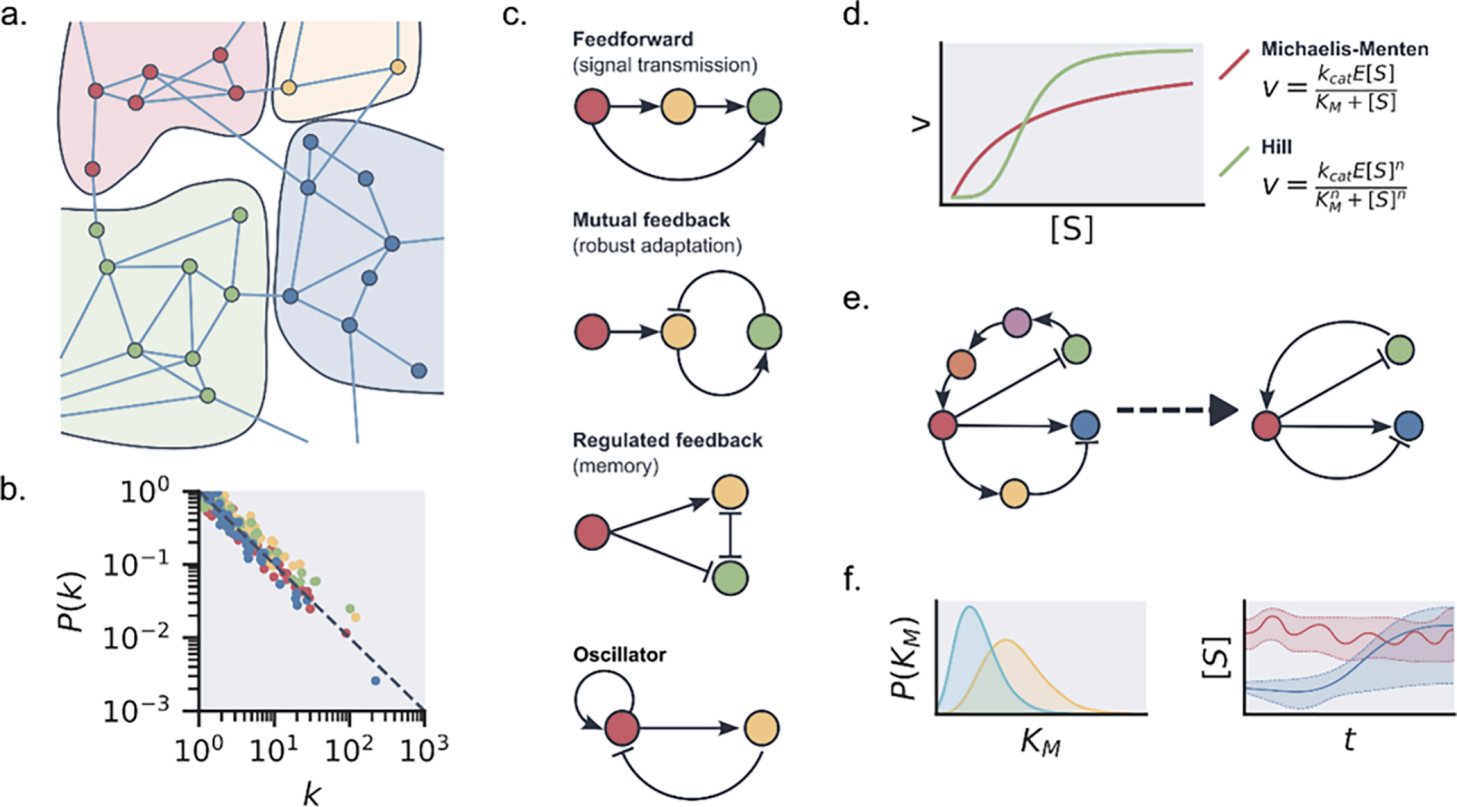
(a) A schematic representation of the modular organization of enzymatic networks in the cell. Strongly connected modules with their own specific functions share limited connections to different modules. In this network representation, enzymes are represented by nodes and substrates by edges. (b) An example of typical scaling relationships found in the connectedness of enzymes. Here, dots represent types of enzymes, with colors denoting different modules. *k* represents the number of connections an enzyme has in the ERN, while *P*(*k*) represents the frequency of that number of connections occurring. Inside cellular ERNs, these follow a power-law relationship *P*(*k*) ≈ *k*^–1^. (c) Examples of network motifs often encountered in ERNs. Again, enzymes are represented by nodes and substrates by edges. Edges with arrows indicate a positive interaction (e.g., a substrate that can be used as a reactant), while a flat end indicates an inhibitory effect. (d). Example of the difference in reaction velocity *v* as a function of substrate concentration between an enzyme with Michaelis–Menten (MM) kinetics and Hill-type kinetics with substrate affinity *K*_M_ and turnover number *k*_cat_. (e) (Left) Example schematic of a full ERN where all interactions are included. (Right) A reduced model form where only essential interactions are maintained. (f) (Left) Bayesian analysis results in parameter estimate probability distributions instead of point estimates, allowing for nonsymmetric errors and standard deviations. (Right) Bayesian estimates can be used to generate a full probabilistic picture of possible enzymatic behavior.

At first sight, the enzymatic networks encountered in living systems show an overwhelming complexity. However, statistical network analysis shows that these networks possess a strongly structured topology, where network topology refers to the large-scale logical structure of a network and the statistical properties of its connections.^11^ For example, the full metabolism is divided into strongly connected modules and pathways that each have their own function (a schematic example is shown in Figure 2a).^12,13^ This modularity is reflected in the statistical description of interactions in ERNs. Generally, these are found to follow scaling relationships (Figure 2b).^14−16^ The distribution of reactions per enzyme follows an exponential scaling distribution. This means that many types of enzymes only promote one or a few reactions, while a few enzymes promote many reactions. This characteristic distribution makes the network more robust to failure while remaining efficient. Identifying the most common network topologies for specific functionalities and the most and least prominent enzymes in those networks can lead to insights into plasticity and redundancy features of specific network topologies,^17^ which can be exploited by ERNs to function in a range of different environments.^18^ A downside to this statistical approach to ERNs is that it only gives a large-scale picture, neglecting any heterogeneity introduced by different types of enzyme reactions and dynamics, and does not distinguish between substrates and effector molecules. Additionally, it does not translate well to the analysis of artificial ERNs, as these networks are often too small to warrant a proper statistical treatment.

### 2.2 Network Motifs

While ERN topology analysis can provide us with information on the dynamics of large and complex systems, zooming in to smaller subunits can help us gain a better understanding of how certain properties arise from the combination of just a few enzymes. Motifs are the smallest functional units, meaning that an individual motif is capable of showcasing complex behavior such as oscillations, adaptation and memory, amplification, and filtering.^19^ A classic example is the emergence of ultrasensitivity in a simple network containing a forward and backward reaction catalyzed by two different enzymes and the emergence of adaptation by inhibitory feedback between two different enzymes converting the same substrate, both key motifs in signaling networks. In their classic paper, Goldbeter and Koshland analyzed the kinetics of such systems and explored the control parameters which would show sensitivity.^20,21^ In 1997, Barkai and Leibler showed how robust adaptation results directly from network connectivity.^22^ Milo and co-workers generalized the concept of these small subnetworks and identified network motifs across a range of different networks—repeated patterns of interactions occurring in large and complex networks at a higher rate than a random network with equivalent topology.^23^ Examples of these include simple cascades, positive or negative feedback loops, or bifans (see Figure 2c). Interestingly, network motifs can vary dramatically between different types of networks but may remain largely the same when different networks have the same type of functionality. For example, gene regulation, neurons, and logic circuits often contain feed-forward loops and bifans to enable rapid switch-like behavior, which are different from those found in food webs, electronic circuits used for arithmetic, and social networks. Similarly, in ERNs, different networks with similar behavior are found to often have the same motifs.^24^ This can be clearly seen in the design of enzymatic oscillators, which almost exclusively involve delayed negative feedback loops, although more complex motifs may be involved as well.^25^ More recently, researchers have shown how different large network topologies can lead to the same effective network motifs capable of, for example, homeostasis and how descriptions of larger networks can be reduced to smaller functional motifs.^26^ Network motifs can be used as a starting point for the design of specific behavior, inspired by either behavior shown in already existing ERNs^27,28^ or recreating network motifs not found in ERNs but in other types of networks.

### 2.3 Enzyme Kinetics

Topology and motifs constrain what types of behavior are possible in ERNs, but the kinetics of enzymatic reactions ultimately determine what will happen and how fast. Many enzymatic reactions are traditionally characterized by Michaelis–Menten (MM) kinetics. Michaelis–Menten kinetics assume a two-step mechanism, where the binding of the enzyme to the substrate is reversible and the release of product is not. While a number of assumptions underlie MM kinetics, it is found to be generally applicable even when some of those assumptions do not hold. In the case of cooperative binding, the Michaelis–Menten equation can be extended to the Hill equation (Figure 2d). For multisubstrate enzymes and inhibitory effects, further extensions of the MM equation are possible.

In recent years, further improvements and replacements to MM kinetics have been developed. For example, Piephoff et al. established a generalized form of the Michaelis–Menten equation by explicitly incorporating nonequilibrium effects.^29^ They took into account how different enzyme conformations impact the speed of the reaction and automatically corrected for different types of substrate binding and allosteric effects. Alternatively, Rowher et al. have shown that the reversible Hill equation is a good general approximation for a large variety of mechanisms found in enzyme kinetics.^30^

However, kinetics data for individual enzymes or complete ERNs are often limited. Consequently, the data used to obtain kinetic parameters can be insufficient to obtain correct estimates. To warrant against overfitting of data, care should be taken to choose equations that do not contain an excess of parameters.^31^ This can be checked for by performing sensitivity analyses on each of the parameters in the kinetic model if a large network proves to be partially unidentifiable; a viable reduced-form network description might still be obtained. By determining which interactions and variables are sloppy and subsequently removing those from the network model, a reduced description shows only those enzymes and interactions that influence the network behavior (schematically shown in Figure 2e).^32^ This method has been used effectively to obtain efficient descriptions of cell-signaling networks^33^ but has yet to be used for artificial ERNs.

Alternatively, so-called Bayesian inference techniques can be used for parameter estimation.^34^ Here, one can use prior knowledge, such as the physically allowed range or probabilistic estimates obtained from previous measurements, to constrain parameter estimations and obtain probabilistic uncertainty intervals and predictions (Figure 2f).^35^ Data with non-normal, or unknown, noise distributions can also be incorporated with relative ease by including additional measurement noise terms in the likelihood function. Bayesian inference methods allow for much more accurate quantification of the uncertainties remaining in a model fit.^36^ Hierarchical Bayesian models can be used to correctly combine data from different experiments and measurement techniques, taking into account different accuracies, noise profiles, and different subsets of observable parameters, and can even be used to compare the likelihood of different reaction mechanisms.^37^

More recently, techniques to check the identifiability of parameters in enzymatic networks directly from equations have become available. These require the construction of so-called sensitivity matrices directly from the kinetic models in combination with experimental data.^38^ When parameters are unidentifiable from a single-input experiment alone, further analysis is required to check if changing the experimental conditions can improve parameter identifiability.^39^ More recently, a new approach has been developed to calculate optimal experimental designs for parameter identification.^40^ This method uses pulse patterns for input substrates in flow to decorrelate kinetic parameters and improve identifiability.^246^ Alternatively, work has been done to decompose a full ERN into separate submodules which are individually identifiable.^41^

Choosing the right analysis method to obtain an accurate kinetic model depends on both the nature and the complexity of the system and the goal behind it. If the goal is optimizing for specific reaction products, it requires a different amount of knowledge than when the goal is to achieve a fundamental understanding of all interactions in an ERN. As shown by the recent developments discussed above, structural identifiability analysis and (Bayesian) parameter estimation highlight the importance of proper uncertainty quantification in increasingly large and complex ERNs.

## 3 Complex Nonlinear Behavior in Artificial ERNs

**Figure 3:**
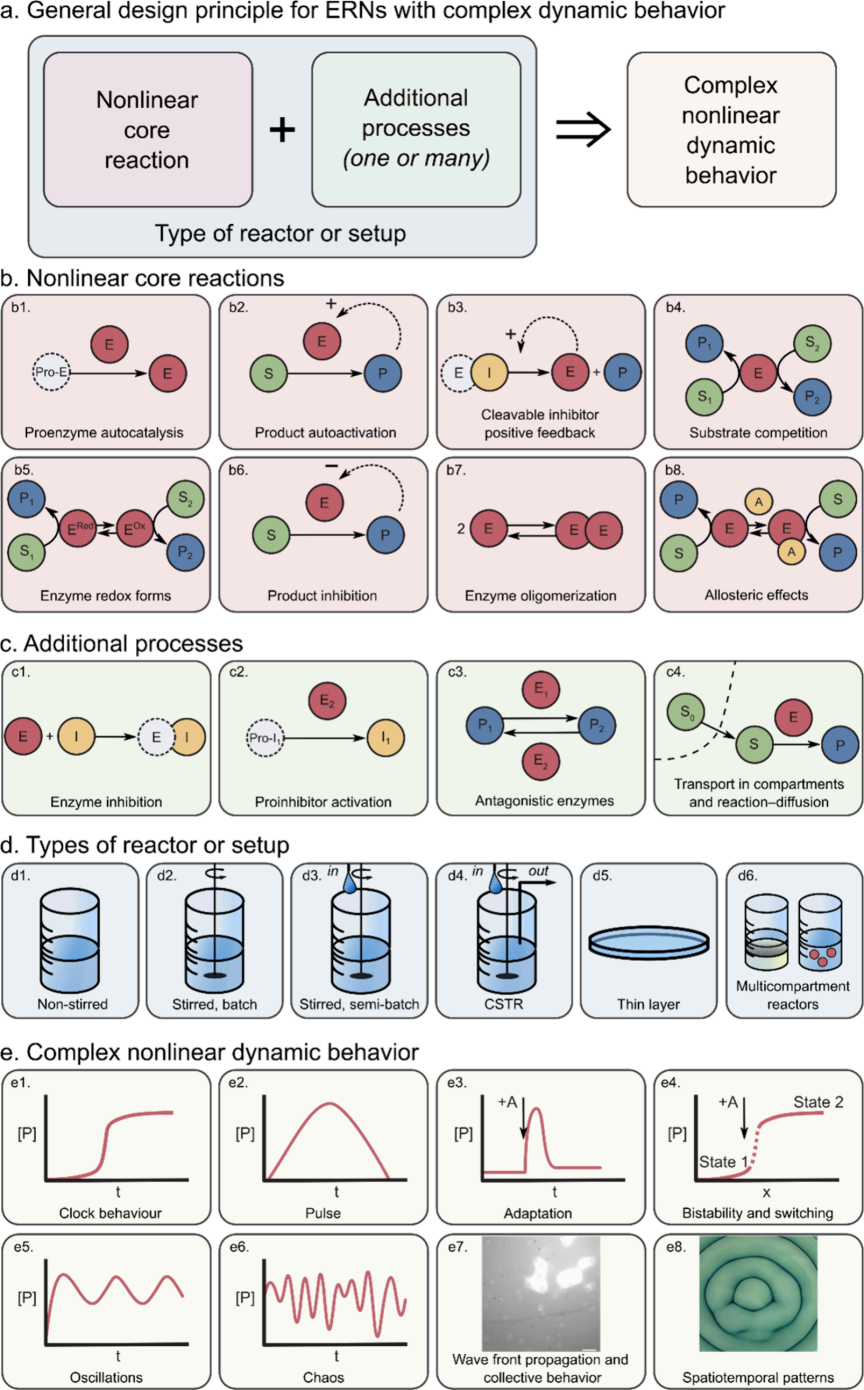
Design of artificial ERNs with complex nonlinear dynamic behavior. (a) General design principle for ERNs with complex dynamic behavior. (b) Central reactions and motifs providing core nonlinearity. (c) Additional processes serving to alter the kinetics of the core motifs and connect different nodes. (d) Types of reactor or setup. (e) Examples of complex nonlinear dynamic behavior available in artificial ERNs. Illustrations: (e7) 50 μm polyacrylamide beads with immobilized urease in a solution containing 5 mM acetic buffer, 50 mM urea, and fluorescent dye SNARF-5,6, scale bar 100 μm, own data; (e8) redox forms of the dye ABTS in the solution containing glucose oxidase (GOx) and horseradish peroxidase (HRP). Adapted with permission from ref (78). Copyright 2018 Springer Nature.

As discussed above, nonlinear dynamics introduced by positive (autocatalytic) or negative (inhibitory) feedback loops are key characteristics of networks exhibiting complex properties. In contrast to the large metabolic and signaling networks found in living systems, synthetic enzymatic networks with complex behavior are typically based on one or just a few feedback reactions. Figure 3a illustrates the general principle behind the design of artificial ERNs with complex nonlinear dynamic behavior: a nonlinear core reaction motif is chosen and combined with additional processes; the actual behavior of this combination is then dependent on the type of reactor that is used. Although the additional processes are typically dependent on the enzymes chosen for the core motif and thus the two are often intertwined, we explicitly separate the two to aid the reader in appreciating the network design and analyzing their dynamics.

The full toolbox of nonlinear core enzymatic reactions described in the literature to date is presented in Figure 3b. The use of autocatalytic core reactions is prominent not only in enzymatic networks but also in organic and DNA networks,^42^ and the design principles of autocatalytic networks were recently reviewed by Semenov and Plasson *et al.*.^43,45^ The exploitation of feedback in the engineering of ERNs was reviewed by Bánsági and Taylor.^44,45^ The three general ways to autocatalysis in ERNs are shown in Figure 3b1–b3. Figure 3b1 shows autocatalysis by self-activation of an inactive zymogen, with the trypsinogen–trypsin (Tg–Tr) pair as a well-known example discussed in detail in section 3.2. Product autoactivation (Figure 3b2) is another very general mechanism with examples mostly involving urease (section 3.1). The cleavable inhibitor positive feedback is relevant for proteolytic networks (see section 3.2) and shown in Figure 3b3. As we discuss later, individual autocatalytic steps are not a general prerequisite to complex behavior, and autocatalysis can also emerge from closed cycles where the separate steps are linear.^242^ The alternative nonlinear cores, namely, those built on substrate competition (Figure 3b4) and switching between enzyme redox forms (Figure 3b5), also deliver nonlinear dynamics, as discussed in section 3.3 using the example of oxidoreductases. Additionally, there are three types of nonlinear cores that are somewhat underexploited. Product inhibition (Figure 3b6), an example of which is the autoinhibitory catalysis of esterase, can be a nonlinear core but has only been found as an additional process in networks comprising a urease core (see section 3.1). The enzyme oligomerization (Figure 3b7) and allosteric effects (Figure 3b8) are well-known ways to achieve nonlinearity, but they have only been demonstrated in theoretical studies and in *in vivo* networks.^20,21^

To achieve controlled complex behavior, a core motif is supplemented and altered by what we call additional processes (Figure 3c). These processes may also serve to connect different network nodes. Inhibition (Figure 3c1) slows down the reaction rate of a certain enzyme, which is an exceptionally useful feedback loop in the case when an inhibitor is produced by another enzyme (Figure 3c2). Effectively, this connects two enzymatic activities that are otherwise independent (see section 3.2 on the trypsin oscillator). Another way to connect enzymatic activities is via the use of antagonistic enzymes (Figure 3c3), with examples mostly on pH-dependent urease networks (section 3.1). Finally, throughout sections 3.1–3.3 we discuss how transport processes enrich modes of dynamic behavior. Transport across membranes, compartment borders, between phases, and simple diffusion (Figure 3c4) add complexity and provide important types of nonlinear dynamics relevant to nature (collective behavior and patterns, Figure 3e7 and 3e8 correspondingly). The influence of transport processes has been recently studied theoretically, highlighting how spatial localization and transport phenomena in nonstirred systems can yield complex nonlinear responses even in fairly simple networks.^46,47^ The transport processes are only relevant in nonstirred systems (Figure 3d1 and 3d5) or in compartmentalized systems (Figure 3d6). Other reactor features presented in Figure 3d can supply reagents and remove products, maintaining the network out of equilibrium (Figure 3d3 and 3d4).

Combinations of the core reactions and additional processes yield a range of possible complex behaviors as shown in Figure 3e. While some types are more common and relatively straightforward to obtain (for instance, clock behavior (Figure 3e1) or pulse-like response (Figure 3e2)), some other types are rare (chaos as shown in Figure 3e6 is, to the best of our knowledge, only obtained in peroxidase–oxidase reaction (section 3.3)).

In order to provide the reader with a readily accessible overview, we grouped the synthetic ERNs with complex nonlinear behavior according to the central enzymatic reaction around which the network is built. For all of the discussed networks, we refer to the motifs they contain and behavior types they produce, as per Figure 3.

### 3.1 Networks Based on the Urea–Urease Reaction

In general, enzymes producing acid or base are a fruitful ground for designing systems with complex dynamics due to the nonlinear kinetics intrinsic to pH-reactive systems.^48−50^ A simple and well-studied enzymatic network that produces nonlinear behavior is the urea–urease network. The enzyme urease (Ur) is found in many bacteria and plants. It catalyzes the hydrolysis of urea into carbon dioxide and ammonia, resulting in an increase in pH. The enzyme has a standard bell-shaped activity–pH profile with an optimum around 7.5.^51^ When starting from a lower pH (usually buffers of pH 3.5–5.0 with low buffer capacity), the enzyme basifies the solution, thereby overcoming the buffer and increasing its own activity, up to pH 7.5. As the reaction proceeds further to even higher pH values, the Ur activity drops again and the final pH of about 8.5 is that of ammonia/ammonium buffer. This process creates an autocatalytic sigmoidal signature in pH vs time, and the time of the steep transition from low to high pH state is addressed in the literature as “clock time” (Figure 3e1).^51,52^ The increase in Ur activity due to the Ur reaction is an example of product autoactivation (Figure 3b2).

Compartmentalization and coupling to diffusion enrich the range of complex behaviors obtained in Ur networks. Effectively, the simple combination of the Ur nonlinear core (Figure 3b2) with transport phenomena (Figure 3c4) yields five types of complex behavior (Figure 3e1–e7), particularly because diffusion coefficients for H^+^ and other species are very different. Ur-loaded millimeter-sized particles exhibit quorum-sensing-like autoactivation (similarly to Figure 3e7), which is a consequence of autocatalysis and diffusion front propagation.^53^ Miele et al. demonstrated the collective synchronized behavior of Ur-loaded microvesicles.^52^ Muzika et al. showed sustained oscillations in a flow reactor with differential influx of urea and H^+^,^54^ building on earlier work on bistability,^55^ and noise-induced irregular oscillatory switching.^56^ Oscillations were also obtained in a system with Ur-loaded lipid vesicles by combining the clock reaction inside of the vesicle with the influx of urea and H^+^ from the bulk phase.^57^ Finally, oscillations in packed arrays of Ur-loaded beads with influx of urea and acid from the bulk solution were predicted theoretically but not yet experimentally proven.^58^

**Figure 4:**
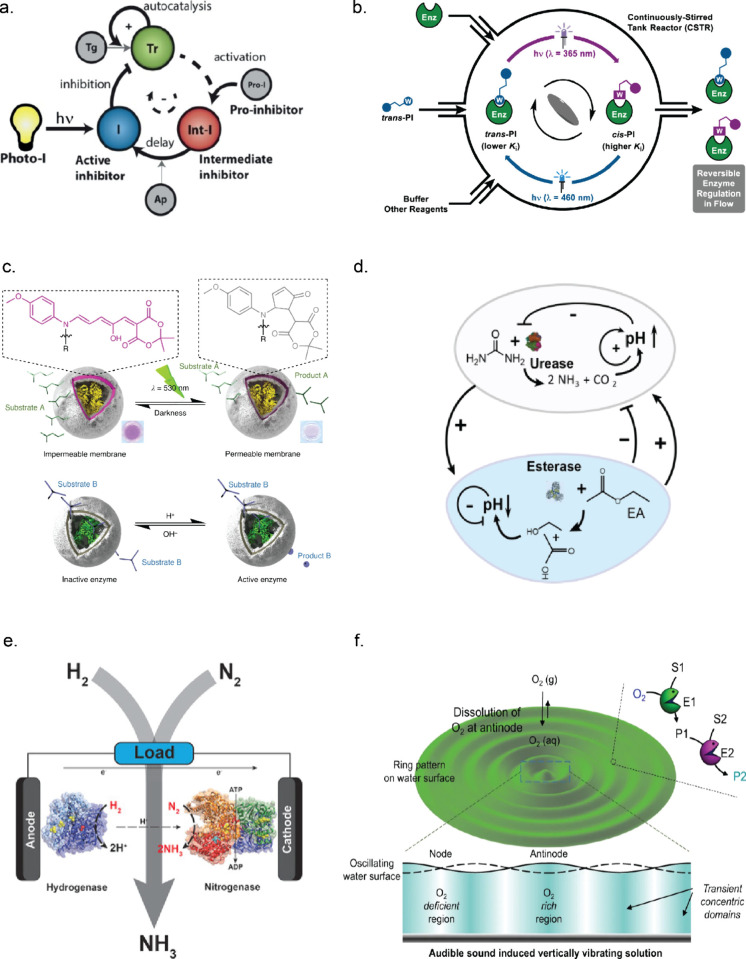
Overview of enzymatic reaction networks that are controlled by different external stimuli. (a) Schematic representation of the trypsin oscillator where the enzyme activity is regulated by a photocleavable inhibitor. Adapted with permission from ref (86). Copyright 2020 John Wiley and Sons. (b) Photoswitchable inhibitor that can reversibly control the enzyme activity in an out-of-equilibrium system using light. Adapted with permission from ref (88). Copyright 2020 licensed under CC 4.0 American Chemical Society. (c) DASA-based polymersomes enable reversible control over the accessibility of substrate toward the active site of an enzyme by light. Adapted with permission from ref (93). Copyright 2022 licensed under CC 4.0 Springer Nature. (d) Ur–Est-based network where Ur increases the pH and Est decreases the pH. Adapted with permission from ref (65). Copyright 2021 John Wiley and sons. (e) Haber–Bosch process where NH_3_ can be generated from H_2_ and N_2_ using enzymes, hydrogenase, and nitrogenase by applying an electrochemical potential. Adapted with permission from ref (106). Copyright 2017 John Wiley and Sons. (f) Cascade network that is spatiotemporally controlled by sound. Adapted with permission from ref (111). Copyright 2022 licensed under CC 4.0 Springer Nature.

An alternative approach to obtain richer dynamic behavior from the urea–urease network is combining it with antagonistic acidifying enzymes (Figure 3c3). Heinen et al. showed that a urea–urease/ester–esterase network produces pulse-like pH responses under batch conditions with, first, a Ur activity-related increase followed by an esterase (Est) activity-induced decrease in the pH (see Figure 4d for network topology). The kinetic profile of the response can be finely tuned by changing the buffer and substrate composition.^59^ Compartmentalization of Ur and Est units in a two-layered gel aided in programming of transient acidic pH flips.^60^ This latter work also addressed the lack of nonlinearity of the Est response (as compared to Ur), which originates in the shape of the Est pH–activity profile and its autoinhibitory nature (Figure 3b6). An alternative acidifying enzyme is glucose oxidase (GOx), converting glucose to δ-gluconolactone, hydrolyzing spontaneously into gluconic acid. Wang achieved dynamic pH switching in response to the addition of urea and glucose in a system with Ur and GOx incorporated in pH-responsive polymersomes.^61^ While Ur is very fast, all of the acidifying enzymes explored thus far exhibited certain limitations on rates. We hypothesize that coupling the urea–urease network to faster acidifying counterplayers would yield more robust dynamic behavior and possibly oscillating systems. The pH transition caused by the Ur-based ERNs can be coupled to a wide range of chemical and physical processes,^62^ such as polymerization and sol–gel transitions,^59,63^ gel growth,^64,65^ and precipitation of CaCO_3_,^66^ that all can serve as handles for functionalities as discussed in section 5.

### 3.2 Networks Based on Proteases

A general route to autocatalysis is to exploit the formation of certain proteases from their inactive precursors (zymogens or proenzymes), as shown in Figure 3b1. Semenov et al. constructed a bienzymatic oscillatory network with a core reaction of trypsin (Tr)-mediated trypsinogen (Tg) activation modified with a delayed negative feedback loop via an aminopeptidase-activated trypsin proinhibitor (combination of motifs Figure 3b1, 3c1, and 3c2; full network topology is depicted in Figure 4a).^67^ Further investigations of this trypsin oscillator included studies on the boundary conditions of the oscillatory regimes using a range of slightly different inhibitors in the negative feedback loop^68^ and synchronization with external temperature oscillations.^69^ Exploiting protease zymogen activation as a core step, Helwig et al. demonstrated adaptation to an external stimulus in an incoherent type 1 feed-forward loop network motif, comprising the chymotrypsinogen–chymotrypsin (Cg–Cr) pair and Tr.^70^ The network responded with a peak to a persistent external stimulus (influx of Tr) and adapted over time, bringing the response Cr activity close to the original baseline, a behavior type in Figure 3e3. The possibility to use autocatalytic trypsinogen activation for signal enhancing was demonstrated in a diffusion-reaction system with trypsin and its inhibitor in a gel.^71^ Finally, Kriukov et al. showed in a Tr–Tg network with an additional inhibition step that the combination of autocatalytic activation and flow leads to hysteresis, and the inhibition can be used to control the network dynamics in a history-dependent manner.^72^

A different and novel type of autoactivation in enzymatic systems was introduced by Pogodaev et al. using slow substrates (cleavable inhibitors) for proteinases Tr, Cr, and elastase (Els), as shown in Figure 3b3.^73^ The reactions started in highly inhibited states, where the slow substrates occupied the active sites of the enzymes. With time, the peptides were cleaved into low-inhibitory fragments, thereby liberating more of the free enzymes, leading to an autocatalytic increase in enzyme activity. It was also demonstrated that in mixtures with Cr, Tr, and their corresponding slow substrates, crosstalk occurred, while Cr and Els remained orthogonal under these conditions, which opens roads to modular construction of modified networks. By converting a cleavable peptide inhibitor into a phosphate-modified proinhibitor, the behavior of the network was coupled to the activity of alkaline phosphatase, which acted as an initiator of the flip response in Cr activity. This is another example of using the motif of proinhibitor activation (Figure 3c1 and 3c2)) to connect behaviors of two enzymes.^73^

### 3.3 Networks Based on Oxidoreductases

In oxidoreductase networks, the nonlinearity often originates from the switching between redox and coordination forms of the enzymes (Figure 3b5).

The earliest example of an *in vitro* enzymatic oscillatory reaction is the peroxidase–oxidase (PO) reaction, first described by Yokota and Yamazaki in 1965.^74^ Oscillations occur during the horseradish peroxidase (HRP)-catalyzed oxidation of NADH (reduced nicotinamide adenine dinucleotide) by oxygen in the presence of additives such as methylene blue and 2,4-dichlorophenol in a semibatch stirred reactor with an influx of NADH and diffusion of oxygen from a gas mixture and no outlet as shown in Figure 3d3. The PO reaction has a complex mechanism comprising multiple redox and coordination forms of the heme cofactor of HRP and very rich dynamics that include normal, period-doubled, and mixed-mode oscillations, quasi-periodicity, and chaos, shown in Figure 3e5 and 3e6.^75,76^ It is hypothesized that many more new dynamic states of the PO reaction are still to be discovered using experimental parameter combinations that go beyond the current regimes, with the pH being an especially important control parameter.^76^ We refer the reader to more specialized literature for further details.^75,77^

Recently, HRP was combined with GOx to produce tunable pulse responses in a stirred reactor (Figure 3e2) and form spatiotemporal patterns (Figure 3e8) in a nonstirred thin layer experiment. Remarkably, this example of complex dynamics is not comprised of individual steps of autocatalytic activation or inhibition, with the nonlinearity originating only in delayed feedback loops and substrate competition (Figure 3b4).^78^ A unique aspect of the behavior of this network is the spontaneous formation of patterns of convection flows when not stirred.

Another enzyme exhibiting autocatalytic kinetics is hydrogenase, a metalloenzyme catalyzing the reaction H_2_ = 2H^+^ + 2e^–^. The mechanism of autocatalysis differs from the simple acid–base activation/deactivation shown for urease and is thought to be connected to redox forms.^79^ The hydrogenase reaction has been observed to produce reaction fronts and oscillations in a thin layer reaction–diffusion experiment via a mechanism that is not yet fully understood.^80^

### 3.4 Summary

In summary, synthetic *in vitro* ERNs with complex behavior have exploited a limited set of core reactions but demonstrated a large range of types of complex behavior. As the recent finding with hydrogenase demonstrates, identifying novel experimental realizations of theoretical network motifs is still a fruitful area of research. Expanding the types of enzymes and combining multiple enzymes into more complex networks are necessary to broaden the scope of accessible network motifs and to realize the theoretical motifs not found in real networks yet. Currently, only two out of seven EC classes of enzymes (oxidoreductases and hydrolases) are used as core reactions in complex ERNs.

An important open question is the design of an ERN which would sustain oscillations under batch conditions (stirred or nonstirred), an enzymatic analogue of the Belousov–Zhabotinsky (BZ) reaction.^247^ The oscillators described in the sections 3.1–3.3 rely on fluxes of reagents (by means of flow reactors or transport processes between compartments). In contrast to the BZ reaction, all enzymatic networks reported thus far consume all “fuel” in a single reaction cycle under well-stirred conditions, thus making it impossible to sustain oscillations in batch. In general and as highlighted by the nonautocatalytic HRP/GOx network example, it is still an open fundamental question what network motifs are required to yield a certain desired type of nonlinear behavior. A strong coupling between experimental and theoretical studies is needed in this regard, especially when incorporating diffusion and other transport phenomena.

## 4 Influence of External Stimuli on ERNs

It is difficult to engineer the properties of complex enzymatic reaction networks as their dynamic output requires all reaction rates to be adjusted to each other, while the formation of byproducts or the emergence of hidden interactions could prevent or disrupt the dynamic behavior of the system.^81^ Fine-tuning rates by controlling the activity of individual enzyme activities is therefore desired. Multiple methods were developed to gain control over enzyme activity by applying an external stimulus such as light, pH, redox potential, heat, magnetic field, and sound.^81−83^ In this section, we will focus on the use of external stimuli to control enzymatic reaction networks that consist of two or more enzymes.

### 4.1 Light

Light irradiation in combination with photocleavable or photoswitchable molecules allows for selective, rapid, and spatiotemporal control over enzyme activities.^81,84,85^

Pogodaev et al. used ultraviolet (UV) irradiation of a photocaged irreversible inhibitor to obtain control over the oscillations of the Tr oscillator discussed in section 3.2 and presented in Figure 4a.^86^ The oscillations were dependent on the duration and timing of UV light, demonstrating that external stimuli can introduce extra complexity without disrupting the rest of the system. However, for many enzymatic systems it is desirable to obtain reversible control over the enzymatic activity. The groups of Feringa, König, and Szymanski have reported many examples where photoresponsive molecules are used to reversibly inhibit specific enzymes upon light irradiation.^83,85,87^ For this, photoswitchable molecules such as azobenzenes, spiropyrans, diarylethenes, and donor–acceptor Stenhouse adducts (DASAs) could be used.^85^

The method to use photoswitchable molecules in combination with light is known as the chromophore-warhead strategy, where one of the two conformations acts as a stronger inhibitor. The warhead, a functional group that is known to inhibit the enzyme, can be covalently attached to a chromophore that can change their 3D structure upon light irradiation.^85^ Using this strategy, Teders et al. recently designed an azobenzene-based inhibitor for Tr and Cr.^88,89^ The activity of these enzymes could be reversibly adjusted under flow conditions upon different light intensity, duration, and wavelength of light, see Figure 4b. These systems exhibited an ultrasensitive response, illustrating potential for using light as an input for controlling complex dynamics in ERNs.

In addition to directly photoswitching molecules that interact with the enzyme (via the active site or allosteric interactions), one could alter the kinetics by controlling the accessibility of the substrate toward the enzyme, as demonstrated by encapsulating enzymes in vesicles or polymersomes and controlling the transport of substrate using light.^90−93^ The latter example, presented in Figure 4c, was based on polymersomes containing a DASA dye in the polymer shell, allowing switching between semipermeable states.^93^ These DASA polymersomes were filled with Est and then mixed with Ur containing polymersomes that were permeable and light insensitive. Upon green light irradiation, the Est–DASA polymersomes became semipermeable, which resulted in the conversion of ethyl acetate into acetic acid by Est. A concomitant pH decrease from pH ≈ 8 to pH ≈ 7 activated urea hydrolysis by Ur, which resulted in a pH rise. The pH increase by Ur resulted in the deprotonation of a pigment that absorbs green light in the protonated form. Therefore, the absorption of green light stopped, and the Est activity could be reactivated.

### 4.2 pH

All enzymes have a specific pH value at which their activity is maximized.^94,95^ In pH-dependent networks, the pH can be altered by addition or generation of acid or base by another enzyme. Often used combinations contain Ur, Est, GOx, or HRP as sources of acid or base.^48,59,96^ As described by Che et al., the addition of chemical fuel can influence the biocatalysis of pH-sensitive polymersomes loaded with HRP or Ur.^97^ The polymersomes shrank in a high-pH buffer due to deprotonation, whereby they became impermeable and thereby inactivate the enzyme. Addition of hydrochloric acid (HCl) and urea (fuel) resulted in a decrease of pH, swelling of the polymersomes, and activation of the enzymes. The depletion of urea resulted in shrinkage of the polymersomes and a decrease in the enzymatic activity. The activation cycle could be repeated multiple times by addition of acid and fuel to create a pH-dependent system. Maity et al. constructed an Ur/Est loop by encapsulating Ur and Est in different hydrogel beads that formed pH fronts.^65^ As described in section 3.1, the conversion of urea by Ur results in a pH increase which is counteracted by the conversion of ethyl acetate into acetic acid by Est, see Figure 4d. The same pH-dependent enzymatic reaction network was used to create enzymatic logic gates.^98^

Wang et al. developed a DNA-based hydrogel that contained GOx, acetylcholine esterase (AchE), and Ur to alter the pH.^99^ GOx and AchE activity resulted in a decrease in pH, while the hydrolysis of urea by Ur increased the pH. Different enzyme compositions allowed control over the pH and thereby the stiffness of the hydrogel. Control over enzymatic batch processes was obtained with acid-producing enzymes and poly(methacrylic acid) (PMAA)-functionalized gold particles. First, these particles were dispersed in the reactor, and reaction-induced pH changes were allowed to take place. When the buffer capacity was exceeded, protonation of the PMAA led to aggregation of the functionalized gold nanoparticles, which could be resuspended upon addition of fresh buffer and substrate.^100^ Instead of changing the pH of the bulk solution, Zhang and co-workers showed that the enzyme kinetics of immobilized enzymes could also be influenced by pH changes in the microenvironment.^95^ The local pH around cytochrome *C*, which is most active under acidic conditions, could be lowered by immobilization on negatively charged high-density polyelectrolytes that can attract ions of opposite charge. The throughput of a cascade with immobilized cytochrome *C* and d-amino acid oxidase was increased 10-fold by lowering the local pH of cytochrome *C* in an alkaline environment where d-amino acid oxidase is most active.

### 4.3 Electrochemical Control

The redox potential can be used as an external stimulus to control the activity of enzymes.^101−103^ Mallawarachchi et al. showed that the accessibility of the substrate toward the active site of hexokinase (HK) entrapped in an electroresponsive hydrogel was altered upon application of an electrochemical potential due to the reversible contraction and expansion of the hydrogel.^104^ The reaction kinetics of enzymes can also be modified by trapping enzymatic cascades in a porous conducting metal oxide electrode material. This was done by Morello et al. to reversibly recycle a nicotinamide cofactor that was generated with an enzymatic cascade consisting of ferredoxin NADP^+^ reductase, l-malate NADP^+^ oxidoreductase, fumarase, l-aspartate ammonia-lyase, and carbonic anhydrase.^105^ It was shown that the reaction direction and rate of ferrodoxin NADP^+^ reductase could be regulated based on the electrochemical potential which allowed control over the synthesis of aspartic acid from pyruvic acid or the reverse reaction. Furthermore, Milton et al. used a combination of nitrogenase and hydrogenase enzymes to electrify the production of NH_3_ from N_2_ and H_2_, which is depicted in Figure 4e. In this work, methyl viologen was used as an electron transfer agent between the enzymes and the electrodes to produce NH_3_ and an electrical current at the same time.^106^

### 4.4 Heat

Heat can be used as a parameter to influence the dynamics of ERNs since the rate constants of enzymes are dependent on temperature.^107,108^ As mentioned in section 3.2, Maguire et al. studied the influence of temperature on the Tr oscillator. They investigated the effect of temperature perturbations under conditions close to the so-called tipping point, the boundary between the oscillatory and the steady state regime.^68^ Close to the boundaries of the stable oscillatory regime (between 15 and 38 °C), sensitivity to short temperature perturbations increased, causing the recovery time to stable oscillations to increase as well. In another study, they investigated the use of small temperature oscillations to regulate the periodicity of the Tr oscillator.^69^ For temperature oscillations with an amplitude of just 3 °C, the periodicity of stable oscillations could be shifted to match the externally induced periodicity through a process known as phase locking or synchronization. Outside the phase-locking regime, quasi-periodic behavior was observed. Since the temperature can have a significant influence on the dynamic behavior of a network, a method to locally adjust the temperature was developed by Zhang et al. This involved the embedding of enzymes on platinum nanoparticles decorated with thermoresponsive copolymers, allowing local heating by irradiation with near-infrared light. The polymer–enzyme hybrids formed aggregates in solution below a certain temperature and disassembled above that temperature, thereby adjusting the accessibility of the substrate toward the active site of the enzyme. In general, the formation of aggregates resulted in a decreased enzymatic activity compared to the disassembled state of the particles.^108^ Thermoresponsive polymers were also used by Gobbo and co-workers to reversibly regulate enzyme activity by contractions of a polymer–protein-based protocell upon temperature changes.^109^

### 4.5 Other Control Factors

The Katz group presented an enzymatic system where a magnetic field was used to induce local pH changes, yielding reversible control over the enzyme activity.^110^ Here, two types of enzyme-modified magnetic nanoparticles that contained amyloglucosidase (AMG) and Ur or Est were used. Upon applying a magnetic field, the nanoparticles with AMG and Ur displayed aggregation. Due to the aggregation, the enzymes were in closer proximity to each other and therefore showed higher enzyme activity. The AMG activity was monitored by a cascade reaction with GOx and HRP.

Furthermore, sound can be used as an external stimulus to control the dynamics of enzymatic cascades. Gradients and patterns of vibrations in an aqueous medium form upon application of sound, see Figure 4f. Dhasaiyan et al. found that at the minima of the vibration, gas dissolution was lower than that at the maxima of the vibration. This was exploited in an enzymatic cascade dependent on dissolved oxygen. First, GOx-FAD was reduced to GOx-FADH_2_ by glucose consumption. The dissolved oxygen was used to produce H_2_O_2_ that can be used by HRP to oxidize ABTS (2,2′-azino-bis(3-ethylbenzothiazoline-6-sulfonic acid). ABTS is a colorful compound that was used to visualize gradients in the enzyme activity of the cascade, see Figure 4f.^111^ Please note, the compartmentalization of enzymes or the immobilization of enzymes on different types of support can enhance the reaction rate and therefore the total output of an enzymatic cascade. For more information about compartmentalization,^120−122^ in DNA nanocages,^123−128^ immobilization in metal organic frameworks (MOFs),^129−133^ or immobilization of enzymes on other supports,^112−119^ we would refer to other reviews.

## 5 Designing “Life-Like” Systems Using ERNs

In previous sections, we discussed how ERNs can exhibit intricate, complex features such as feedback loops, bistability, ultrasensitivity, or adaptive behavior. Employing these complex features of enzyme-catalyzed reactions to trigger morphological transformations and modulate the physical properties could lead to the development of so-called life-like systems. In this section, we will delve into specific examples that highlight the design of complex systems utilizing enzymatic networks.

### 5.1 Dynamic Materials

**Figure 5:**
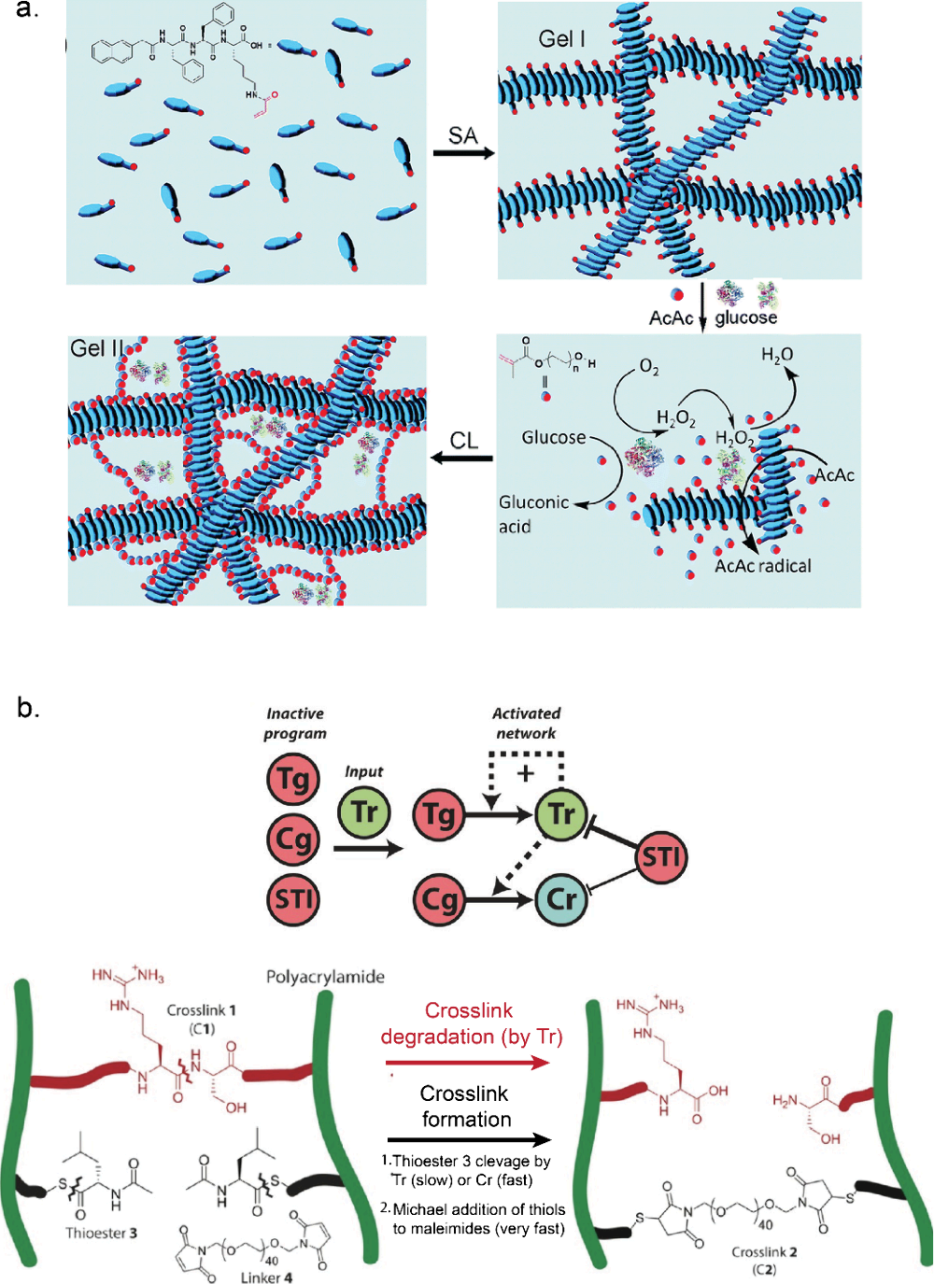
Overview of dynamic materials designed with enzymatic reaction networks. (a) Enzymatic polymerization of hydrogels. Adapted with permission from ref (140). Copyright 2016 licensed under CC 3.0 Royal Society of Chemistry. (b) Schematic representation of cross-link degradation of a hydrogel controlled by an enzymatic network. Adapted with permission from ref (146). Copyright 2017 John Wiley and sons.

Enzyme-catalyzed reactions typically occur under mild conditions and show high substrate specificity, making enzymes an attractive choice for the design of new dynamic materials. To make the link to materials, researchers studied the influence of enzymatic reactions on polymer chains in order to induce self-assembly or disassembly or other morphological reorganizations.^134,135^ For example, Amir et al. reported the use of acid phosphatase to cleave phosphate moieties of hydrophilic monomers, resulting in the formation of an amphiphilic diblock copolymer of polyethylene glycol (PEG) and poly(4-hydroxystyrene) that assembled into spherical micelles.^136^ In another example, the autocatalytic nature of urea-Ur was harnessed to control base-catalyzed thiol-Michael addition reaction for time-lapse hydrogelation.^63^ Enzyme-catalyzed reactions have been utilized to initiate polymerization of monomeric building blocks.^137,138^ Mao et al. used acid phosphatase and GOx to make hydrogels out of supramolecular and polymeric networks.^139^ A similar strategy was later implemented to design printable hydrogels using GOx and HRP.^140^ In this study, an acrylic acid-modified hydrogelator (NapFFK-acrylic acid) was used as a monomer, undergoing self-assembly to form hydrogels (gel-I, Figure 5a). GOx converted glucose to gluconic acid and subsequently reduced O_2_ to H_2_O_2_. Next, HRP utilized H_2_O_2_ to catalyze the formation of acetylacetone (AcAc) radicals via oxidation of AcAc. These radicals initiated polymerization of poly(ethylene glycol) methacrylate (PEGMA) with acrylic-modified hydrogelators, resulting in a cross-linked hydrogel (gel-II, Figure 5a). Gel-II showed very good mechanical properties with an approximately 16 times higher storage modulus compared to gel-I (without cross-linking). Klemperer et al. utilized a similar strategy to design cytocompatible bioinks with interpenetrating polymer network (IPN) formed under mild and aerobic conditions.^138^

Moreover, enzyme-mediated de-crosslinking or cleavage has been exploited to install stimuli responsiveness in the presence of enzymes. Hydrogels containing enzyme-cleavable groups were used for designing responsive materials susceptible to enzymes like proteases, lipase, Est, and phosphatase activities.^141−144^ Yang et al. demonstrated for the first time an enzyme-regulated reversible gel–sol transformation. They utilized kinase–phosphatase switch to regulate assembly of peptide-based hydrogelator (Nap-FFGEY). The addition of kinase and adenosine 5′-triphosphate (ATP) led to phosphorylation of hydrogelator, disrupting self-assembly, while phosphatase reversed this process, restoring the self-assembly property.^141^ Interestingly, micelles formed from polymer–peptide block copolymers modified with substrates for different enzymes (protein kinase A (PKA), protein phosphatase-1 (PP1), and matrix-metalloproteinases MMP-2 and MMP-9) showed different morphologies and aggregation behavior upon enzymatic reactions.^145^ In addition, Postma et al. reported a systematic approach in designing adaptive matter by integrating reaction networks with materials.^146^ They designed a polyacrylamide (PAAm)-based hydrogel containing two orthogonal types of cross-links, which could either be enzymatically degraded or formed (Figure 5b). Tr rapidly cleaved the first cross-links, thus forming a liquid phase. Simultaneously, Tr slowly cleaved a copolymerized thioester (cross-link precursor), and these newly exposed thiol groups reacted rapidly with linker (poly(ethylene glycol)-bis-maleimide) to form a new gel. Upon addition of Tg and Cg, Tr was formed autocatalytically from Tg and thus speeding up the degradation and activation processes of the first and second cross-links, respectively. Tr also rapidly converted Cg into Cr, which induced even faster cleavage of copolymerized thioester. Finally, to set a threshold for activating the entire network, soybean trypsin inhibitor (STI) protein was introduced, which deactivated the enzymatic network by inhibiting Tr activity (Figure 5b).

Enzyme-catalyzed reaction products can also be used as triggering stimuli which influence polymeric assemblies to exhibit dynamic functions. For example, oxidases like GOx, sarcosine oxidase (SOx), choline oxidase (COx), and urate oxidase (UOx) generate H_2_O_2_ upon oxidation of their substrates. Hydrogels containing both hydrolases (AchE) and oxidases displayed a gel–sol transition in the presence of hydrolase substrate (acetylcholine) by the cascade generation of H_2_O_2_.^147^ In earlier sections, we have discussed how enzymes like Ur, GOx, and Est are capable of controlling the pH of the system environment. Walther and co-workers have demonstrated how enzyme-mediated pH feedback can be introduced to design stimuli-responsive smart materials. For example, Heuser et al. integrated Ur-mediated pH feedback to a pH-responsive photonic gel composed of polystyrene-*b*-poly(2-vinylpyridine) (PS-*b*-P2VP) diblock copolymers to control swelling of the photonic hydrogel, which in turn controls transient optical reflection.^148^ Moreover, enzyme-mediated pH change was used to design temporal materials where gelation is controlled by a change in pH via enzymatic reactions.^59,65^ To demonstrate pH-dependent transient gelation, Heinen et al. used a hybrid hydrogel made of DNA-containing polyacrylamide copolymer with pH-responsive DNA cross-linker (i-motif). This pH-responsive unit formed a tetraplex intramolecularly at low pH and as a result did not take part in cross-linking (sol state). With increasing pH, the unreactive tetraplexes unfold and induce hybridization with the copolymerized strand, resulting in a gel state. A sol–gel–sol transition was observed by the antagonistic effect of Ur-mediated pH increase and pH decrease by Est.^59^ In short, enzyme-catalyzed chemical reactions have a diverse range of applications, from enabling cross-linking polymerization under mild conditions to directly controlling material properties to install responsiveness.

### 5.2 Enzyme-Powered Motile Systems

**Figure 6:**
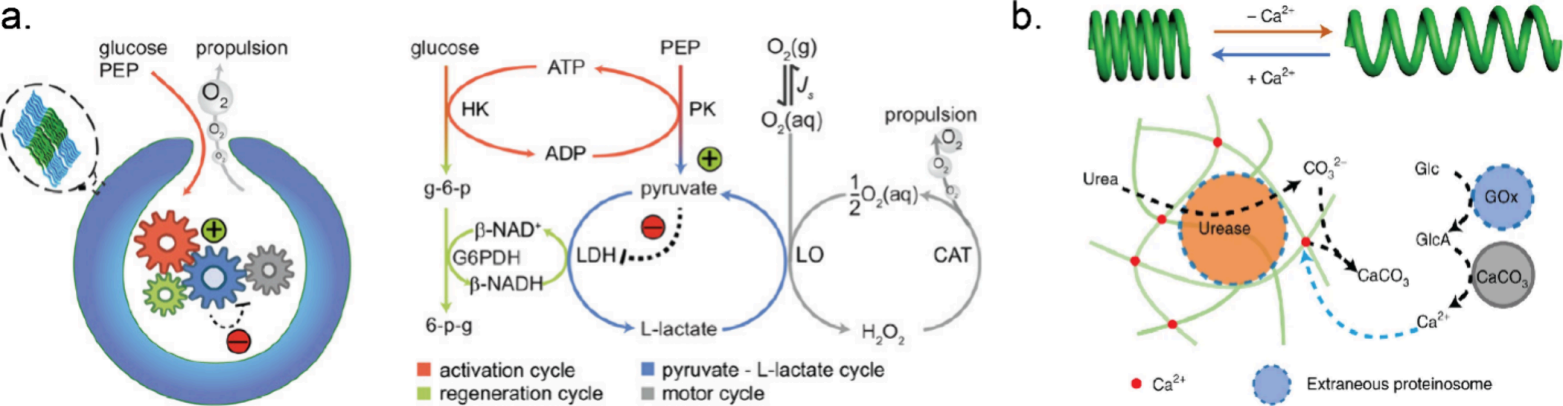
Overview of enzyme-powered motile systems designed with enzymatic reaction networks. (a) Self-propulsion of stomatocytes by generating oxygen from glucose using an encapsulated metabolic enzymatic network. Adapted with permission from ref (162). Copyright 2022 American Chemical Society. (b) Illustration demonstrating expansion and contraction of a spring made of calcium alginate by antagonistic interaction of two enzymes, i.e., Ur and GOx. Adapted with permission from ref (169). Copyright 2021 Springer Nature.

The inspiration for designing motile systems stems from various biological motor proteins which operate via conversion of chemical energy into mechanical motion by the hydrolysis of ATP. Besides these motor proteins, enzymes have shown increased diffusion during substrate turnover and can impart forces (ca. 10 pN) quite comparable with biological motor proteins.^149,150^ In addition, an asymmetric distribution of reactant and product molecules during enzyme catalysis can create local gradients of concentration or an electrical field, which can lead to enhanced diffusion of enzymes.^151^ However, active movement of enzymes in the presence of substrates is part of an ongoing debate, and we refer to other literature sources for a more thorough discussion.^152,153^ A number of research groups have worked on endowing small micro/nanosized particles with enzymes in order to create motile systems.^154−156^ These particles achieved motility by enzyme-catalyzed chemical reactions. For example, Ur can show ionic self-diffusiophoresis due to the generation of a local electric field from diffusivity differences between the oppositely charged ions (NH_4_^+^ and CO_3_^2–^) formed from urea.^157^ Ur has been extensively used as a catalytic engine to design self-propelling motors.^158−160^ On the other hand, oxygen bubble generation from H_2_O_2_ by Cat or a pair of enzymes like GOx/Cat can propel microswimmers opposite to bubble formation.^161,162^ One promising system is based on stomatocytes, bowl-shaped particles formed by deformation of polymersomes by osmotic pressure. During the deformation process, enzymes can be encapsulated in the nanocavity that is formed when the shell folds in on itself, and the enzymatic activity of the encapsulated enzymes provides a force that can propel these stomatocytes.^163^ For example, a metabolic network of six enzymes was compartmentalized in stomatocytes, which was responsible for converting glucose (fuel) into motion (Figure 6a). This network started with an ATP-mediated activation module containing HK and pyruvate kinase with phosphoenolpyruvate (PEP, phosphate donor) and glucose (energy source). Pyruvate (the product of the first cycle) triggered the pyruvate–l-lactate cycle where l-lactate dehydrogenase (LDH) consumed pyruvate. But, l-lactate oxidase (LO) catalyzed the reverse reaction. NADH was regenerated by the conversion of glucose-6-phosphate by glucose-6-phosphate dehydrogenase (G6PDH) into 6-phosphogluconolactone. This resulted in net H_2_O_2_ production, which was converted to molecular oxygen by Cat.^164^ Here, ATP determined the concentration of NADH, thus regulating the entire network. In another example, amyloid microphases loaded with alcohol dehydrogenase (ADH), SOx, and Cat exhibited microscopic motility. These self-assembled structures contained imidazole moieties, which hydrolyzed the 6-methoxy naphthyl alcohol ester of *N*-methyl glycine (starting substrate) to 6-methoxynaphthyl alcohol and *N*-methyl glycine. Subsequently, ADH converted 6-methoxynaphthyl alcohol to 6-methoxynaphthaldehyde (a fluorescent compound), while the cascade of SOx and Cat utilized *N*-methyl glycine to generate oxygen bubbles, responsible for the observed motility.^165^ Increased diffusive movement of free enzymes and enzyme-coated particles toward higher substrate concentration has been reported and extensively studied.^150,166,167^ The Wilson group designed Cat-powered PLGA (poly(lactic-*co*-glycolic acid) micromotors as chemotactic drug delivery vehicles, which can follow the H_2_O_2_ gradient produced by macrophage cells.^168^ Joseph et al. demonstrated how coupling enzyme cascades (GOx and Cat) with asymmetric polymeric vehicles could be used to design potential chemotactic drug delivery vehicles for crossing the blood–brain barrier.^169^

Aside from enzyme-powered self-propulsion, oscillatory movement was achieved in self-assembled organoclay/DNA semipermeable microcapsules containing Cat and GOx. In the presence of H_2_O_2_, Cat produced oxygen bubbles which were taken up by GOx in the presence of glucose. The generation and consumption of oxygen bubbles was responsible for sustained oscillatory movement. The system was able to achieve multiple oscillations under a continuous flow of glucose and H_2_O_2_.^170^ In other recent work by the same group, the interaction between protocells and their environment was explored by designing microactuators capable of chemically induced spring-like compression and relaxation.^171^ Helical filaments of calcium alginate containing Ur-loaded proteinosomes were fabricated using microfluidics. When these coiled hydrogels were exposed to urea, carbonate ions were produced and started chelating the calcium ions (Ca^2+^) from the hydrogel cross-links. This Ur-mediated removal of Ca^2+^ ions led to the release of the stored elastic potential energy, which in turn induced extension of the helical filaments. To contract these extended filaments, they were exposed to proteinosomes containing GOx. In the presence of glucose, gluconic acid was produced, which dissolves CaCO_3_ and releases Ca^2+^. This protocell-mediated Ca^2+^ flux in turn restored cross-links in the calcium alginate matrix which led to a slow retraction of the extended filaments (Figure 6b). These examples highlight current endeavors to utilize enzymes to control motility of artificial systems, which can be useful in designing intelligent sensors and novel therapeutic vehicles.^189^

### 5.3 Communication in Compartmentalized Bioreactors

**Figure 7:**
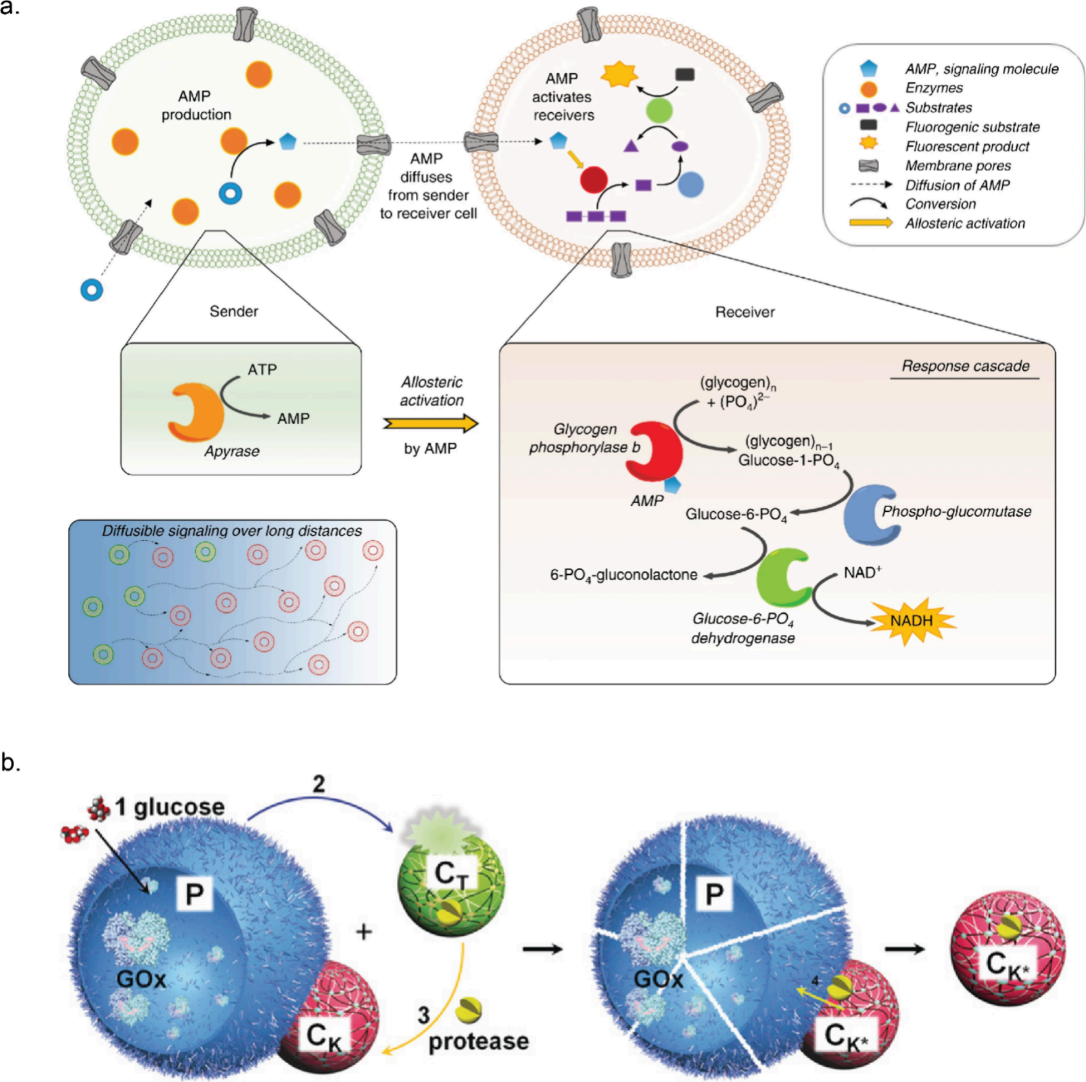
Overview of communication in compartmentalized bioreactors facilitated by enzymatic reaction networks. (a) Communication between sender and receiver GUVs where the sender produced AMP which allosterically activated ERN within receiver GUVs. Adapted with permission from ref (179). Copyright 2020 licensed under CC 4.0 Springer Nature. (b) Cartoon representation of artificial response–retaliation behavior among protocell communities. In the presence of glucose, GOx containing protease-sensitive proteinosome (P) released acid (2) which disassembled pH-sensitive protease containing coacervate (C_T_) and released protease (3). Coacervate C_K_ recaptured protease and eventually destroyed P. Adapted with permission from ref (175). Copyright 2019 John Wiley and sons.

Compartmentalization is an effective strategy to segregate enzymes catalyzing sequential reactions and can be helpful to increase productivity by concentrating substrates and avoiding unwanted side reactions.^120,172,173^ This spatial segregation, coupled with interactions between compartments with distinct chemistries, can further lead to the emergence of life-like properties in artificial systems like predator–prey behavior,^174−176^ chemical signaling,^177−181^ and chemostructural feedbacks.^171,182^ In this context, Elani et al. designed a multicompartment vesicle-based platform to spatially segregate reaction processes where chemical signals can transverse from one compartment to another. A three step cascade by lactase, GOx and HRP was conducted where each enzyme catalyzed step was isolated in distinct compartment.^183^ Wang et al. developed methodologies to design microarrays of giant unilamellar lipid vesicles (GUVs) containing GOx and HRP to facilitate controlled chemical signaling in the presence of melittin, a pore-forming peptide.^178^ Buddingh’ et al. demonstrated chemical communication between giant vesicles through allosteric signal amplification. They used glycogen phosphorylase b (GPb) which switched to a high-activity state upon binding adenosine 5′-monophosphate (AMP). The sender vesicle contained apyrase which produced AMP in the presence of ATP (Figure 7a). The produced AMP diffused to the receiver vesicle, where a small ERN comprising phosphoglucomutase (PMG), G6PDH, GPb, and glycogen was compartmentalized. As AMP acts as allosteric activator of GPb, it induced conversion of glycogen to glucose-1-phosphate, followed by the conversion of glucose-1-phosphate to glucose-6-phosphate by PMG, and subsequent generation of NADH by G6PDH (Figure 7a). This allosteric amplification of the initial weak signal from the sender vesicles, achieved through GPb activation, resulted in a robust response (high NADH output) in the receiver vesicles. Remarkably, the strong signal amplification engineered into this pathway allowed long distance (5 mm) communication between senders and receiver vesicles.^179^

Qiao et al. achieved artificial response–retaliation behavior using ternary protocell populations. These populations included proteinase K-sensitive GOx-containing proteinosomes (P) that released H^+^ in the presence of glucose.^175^ These also consisted of small pH-sensitive proteinase K-containing polypeptide (poly-d-lysine)/adenosine 5′-diphosphate (ADP) coacervates (C_T_) and pH-resistant positively charged polymer (poly(diallyldimethylammonium chloride), PDDA)/polysaccharide (dextransulfate, DS) coacervate droplets (C_K_) that adhered to negatively charged proteinosomes via electrostatic interactions (Figure 7b). Proteinase K was initially sequestered in C_T_ coacervates, but the addition of glucose led to gluconic acid release from P, lowering the pH and causing disassembly of the coacervates C_T_. The released proteinase K was then sequestered by the proteinosome-adherent coacervates C_K_. Finally, proteinosomes were destroyed by proteinase K, and only C_K_ remained. Expanding on the idea of communication between compartments, Chakraborty et al. demonstrated how a bioluminescent signal triggered prey–predator behavior in GUVs. Renillaluciferase (RLuc) containing GUVs (sender) produced blue light in the presence of coelenterazine, activating iLID and Nano proteins in the outer membranes of both sender and receiver GUVs. The interaction between iLID and Nano under blue light mediated the adhesion between the GUVs. GUV–GUV adhesion allowed transfer of Ca^2+^ from the sender to the receiver through unblocked α-hemolysin (α-HL) pores. Ca^2+^ activation of phospholipase A2 (PLA2) in prey GUVs led to phospholipid cleavage and collapse of receiver GUVs.^176^ A recent study from the same group showcased bidirectional communication in GUVs through similar chemiluminescence-triggered adhesion, enabling exchange of H_2_O_2_ from the sender and Ca^2+^ from the receiver. Interestingly, GUVs separated when the signaling molecule production ceased.^180^ Liu and Zhang et al. designed a three-layer tubular prototissue comprising concentrically arranged agarose hydrogel layers containing GOx, HRP, and CAT containing coacervates as the outer, middle, and inner layers, respectively. Glucose and hydroxyurea were added specifically to the exterior side of the model prototissue. Through inward diffusion, glucose was first processed by GOx containing a hydrogel layer, leading to H_2_O_2_ production. HRP in the middle layer converted hydroxyurea in the presence of H_2_O_2_ into nitric oxide (NO). Excess H_2_O_2_ was then consumed by Cat in the final layer, such that NO became the main output, which was further used to inhibit blood coagulation in samples located within the device’s internal lumen.^184^

Interestingly, enzymes fixed in a microchamber can act as “chemical pumps” in the presence of specific substrates. Density differences between the substrates and the enzyme-catalyzed products give rise to solutal buoyancy, in turn generating convective flows of the enclosed fluids.^185,186^ This system can be understood as follows: when the density of the enzyme-catalyzed products exceeds that of the reactants, the fluid becomes denser, sliding down and away from the patch like an outward pump. Conversely, if the product density is lower, the fluid rises up, falls back down due to convective flows, and moves toward the enzyme patch, forming an inward pump. The fluid flow velocity is dependent on the enzymatic reaction rates. These micropumps have been utilized as proof-of-concept delivery vehicles to transport small molecules like insulin in the presence of a glucose stimulus or act as sensors to detect toxins that hamper enzymatic reactions.^185,187^ Furthermore, simulations on flexible sheets with two enzyme patches have revealed complex motility behaviors. The first patch produces substrates for the second, while the products from the second patch inhibit the enzymes in the first patch. The spatial separation between these two enzyme patches led to the time delay required for chemical oscillations in the system. The resulting fluid flow induced oscillatory mechanical deformation, displaying diverse motility behaviors.^188^ These strategies utilizing ERNs to enable communication between spatially segregated bioreactors are crucial for designing systems with diverse life-like applications.

## 6 Cell-Free Synthesis

Cell-free biosynthesis is a rapidly growing field in which complex biochemical pathways are assembled *in vitro*. The aim of this field is to better understand how such pathways function and how they can be manipulated. The reconstruction of these pathways *in vitro* also provides a route to the synthesis of valuable organic molecules using environmentally friendly processes and without the need for culturing organisms. Reaction networks carried out in cell-free environments can, in principle, be much better controlled by optimizing the pH, temperature, and enzyme loading. Furthermore, competition with other enzymatic pathways is excluded, and enzymes produced in various organisms can be combined into a single pathway.^190^ In this section, we will focus on the biochemical pathways that consist of multiple enzymes. For the two-step enzyme cascades, we refer the reader to the reviews in refs (172) and (191−193) and specifically for enzymatic cofactor regeneration to the reviews in refs (194) and (195). Importantly, only cell-free synthesis of small molecules is covered in this review; cell-free protein synthesis has been the topic of several reviews.^196−198^

### 6.1 Sugars as Substrates

**Figure 8:**
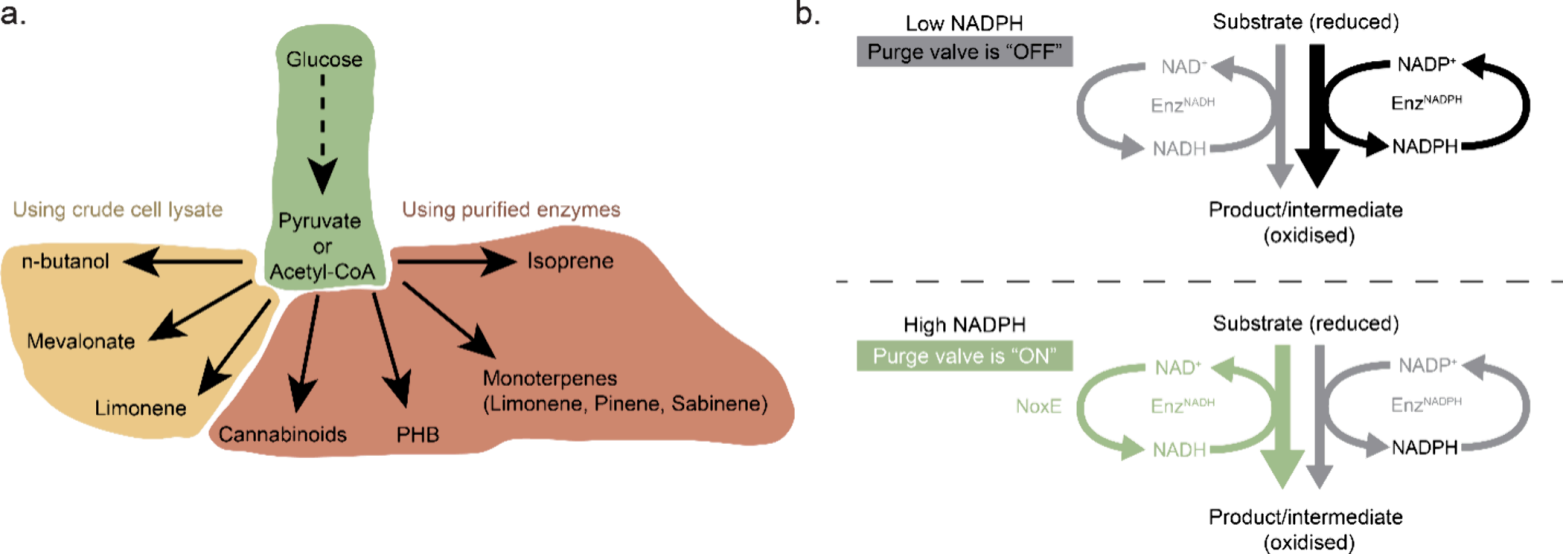
(a) A diagram of low-cost substrate glucose being converted into pyruvate or acetyl-CoA as an intermediate and then depending on the selected pathway to isoprene,^199^ monoterpenes,^200^ PHB,^202^ or cannabinoids^203^ using purified enzymes (in orange) or to *n*-butanol,^205^ mevalonate,^206^ or limonene^208^ using crude cell lysate (in yellow). (b) The purge valve concept, where the purge valve is “OFF” when there is low NADPH concentration and “ON” at high NADPH concentration. Adapted with permission from ref (201). Copyright 2014 Springer Nature.

Bowie’s group demonstrated how complex enzymatic chemical reaction networks can be used to synthesize valuable molecules starting from simple substrates such as glucose or phosphoenolpyruvate (PEP) (Figure 8a). All reactions were performed using purified enzymes in batch conditions. One of the first complex enzymatic reaction pathways included isoprene synthesis, which was synthesized from PEP in a combined glycolysis and mevalonate pathway containing in total 12 enzymes.^199^ ADP, NADPH, and coenzyme A (CoA) regeneration cycles were employed to reuse the cofactors and to avoid CoA build up, which was causing inhibition of the forward direction of the mevalonate pathway. After this, the same group showed how a 27-enzyme cell-free system in batch converts glucose into different monoterpenes using glycolysis and mevalonate.^200^ The choice for the final enzyme in the pathway, a limonene synthase, pinene synthase, or limonene synthase-N345A mutant, determined which monoterpene (limonene, pinene, or sabinene) was produced. The whole pathway also included a molecular purge valve (Figure 8b).^201^ Molecular purge valves maintain cofactor balance in the system without the need to perfectly match stoichiometric cofactor formation and carbon consumption. Once there is a NADPH cofactor buildup, the NADP^+^-dependent reductase enzyme is starved of oxidized cofactor and the pathway shuts down. In the meantime, the purge valve is turned on and NAD^+^-dependent reductase and NADH-specific oxidase are activated. In this case, it was used to control the balance of NAD(P)H/NAD(P)^+^ using three enzymes: glyceraldehyde-3-phosphate dehydrogenase (Gap), mutant glyceraldehyde-3-phosphate dehydrogenase (mGap), and NADH oxidase (NoxE). This cascade regenerates NADPH, purges the excess of NADH, and continues generating carbon building blocks for the glycolysis pathway. It was demonstrated that excluding at least one enzyme of the molecular purge valve reduced limonene production drastically, while a system without the NoxE enzyme did not convert any glucose to limonene. Researchers managed to produce all three terpenes starting from 500 mM glucose over 7 days at high titers.

Another example of synthetic pathways producing valuable organics includes the so-called PBG (pentose–bifido–glycolysis) cycle in which the final product is polyhydroxybutyrate (PHB), a bioplastic.^202^ The whole cycle consists of 20 enzymes from three partial pathways: the pentose phosphate pathway, the bifidobacterium shunt, and the glycolysis pathway. This ERN also contained two purge valves to regulate NAD(P)H concentrations as well as a metabolite salvage pathway which allowed erythrose-4-phosphate to re-enter the cycle. Two new enzymes, a G6PDH and a 6-phosphogluconate dehydrogenase (Gnd) mutant, had to be engineered for the purge valve to favor NAD^+^ instead of NADP^+^, as their wild forms do. A batch reaction containing all enzymes was productive for up to 55 h, and only the last enzyme of the cycle—PHB synthase (PhaC)—had to be added again after each 10 h cycle. This was done because the enzyme was covalently linked to the growing end of the PHB product, which meant the enzyme would be removed every time during sampling of the bioplastic out of the reaction vessel to quantitatively measure produced PHB. Valliere et al. constructed an even more complex pathway consisting of 23 enzymes, which was used to synthesize a variety of prenylated molecules, including cannabinoids, from glucose.^203^ In their work, a modified glycolysis module was developed which included a purge valve to balance NADPH concentration and carbon flux. This module was connected to acetyl coenzyme A (acetyl-CoA) and mevalonate modules to form geranyl-pyrophosphate (GPP). From there, different substrates and their complementary enzymes were added to yield various prenylated compounds. Performing this pathway using only purified enzymes is also advantageous as GPP is toxic to cells in medium concentrations.^204^ The original system containing pyruvate dehydrogenase (PDH) had to be modified as it was found that PDH was inhibited by 1,6-dihydroxynaphthalene (1,6-DHN), which is the preferred substrate for wild-type prenyltransferase (NphB) enzyme. Therefore, a PDH bypass system consisting of two additional enzymes to convert pyruvate via acetyl phosphate to acetyl-CoA was introduced. After it was proved that various prenylated aromatic compounds can be generated using prenyl transferases, the authors focused on optimizing cannabinoid production. The results showed that the production rate of the cannabinoid precursor cannabigerolic acid (CBGA) using the primary system reached a not particularly high titer. However, a new mutant CBGA synthase (M23) was designed to produce CBGA using olivetolic acid (OA) without side products, which helped to reach a higher titer. In combination with *in situ* product removal using a flow system, a final CBGA titer was increased even more.

All of these examples demonstrated complex cell-free purified enzymatic reaction networks and possibilities for future industrial applications. Obviously, some important aspects of any industrial application include optimization, cost reduction, and yield increase. The authors proposed employing stable enzymes that could be used for longer periods of time in order to reduce costs, introducing cofactor (ATP/NAD(P)H) regeneration cycles to increase yields,^204^ recycling enzymes, and introducing cheaper purification methods.^202^ Another potential improvement could come from enzyme immobilization, which allows for easier purification and optimization at a cost of potentially reduced enzyme activity upon immobilization.

Instead of using purified enzymes, in some networks it would be desirable to construct ERNs in crude cell lysate. Karim et al. established a cell-free protein synthesis-driven metabolic engineering (CFPS-ME) framework for fast enzyme selection and pathway construction.^205^ As a model system, they chose a CoA-dependent *n*-butanol pathway starting from glucose including 17 steps *in vitro*. For this, the authors combined cell-free metabolic engineering (CFME), where heterologous enzymes are selectively overexpressed, and cell-free protein synthesis (CFPS). First, it was shown that mixing five crude lysates each of them with different selectively overexpressed enzymes via CFME can activate the whole pathway and produce *n*-butanol. Next, CFPS was modularly integrated in CFME to speed up the biosynthesis. Hydroxybutyryl-CoA dehydrogenase from *Clostridia beijerinckii* (Hbd2) lysate was excluded from the mixture of overexpressed enzymes, and instead, the CFPS was performed for Hbd2. As the downstream enzymes could not be activated without their substrates, the pathway would stay inactive until Hbd2 was synthesized. Therefore, first, CFPS of Hbd2 was performed for 3 h, and glucose was added to initiate the *n*-butanol synthesis pathway. Later, a similar experiment was repeated by excluding every other one of the five *n*-butanol pathway enzymes from overexpressed enzyme lysate mix and synthesizing them using CFPS for 3 h. After adding glucose, NAD, and coenzyme-A (CoA) to initiate the CFPS–ME pathway reactions, all variants produced *n*-butanol. This study demonstrated how the CFPS–ME method can be used for fast enzyme screening for ERNs. The same group also demonstrated the synthesis of mevalonate from glucose using enzyme-enriched lysate mixtures^206^ and investigated a pathway from mevalonate to limonene without any optimization.^207^ This research laid the groundwork for a combined study involving a full pathway from glucose to limonene using enzyme-enriched *Escherichia coli* (*E. coli*) lysates requiring in total 20 metabolic steps.^208^ To begin with, the endogenous metabolism in *E. coli* crude cell lysate already consisted of all of the glycolytic enzymes which transformed glucose to acetyl-CoA. This part was then connected to a single extract enriched in three enzymes—acetyl-CoA acetyltransferase (ACAT), hydroxymethylglutaryl-CoA synthase (HMGS), and hydroxymethylglutaryl-CoA reductase (HMGR)—converting acetyl-CoA to mevalonate. Last, it was coupled to a module of six extracts each enriched in a single enzyme using heterologous overexpression *in vivo*—mevalonate kinase (MK), phosphomevalonate kinase (PMK), pyrophosphomevalonate decarboxylase (PMD), isopentenyl pyrophosphate isomerase (IDI), geranyl diphosphate synthase (GPPS), and limonene synthase (LS). After additional experiments and optimization, the full pathway produced final molecule limonene.

Cell-free biosynthesis points out attractive possibilities of performing enzymatic synthesis and creating complex ERNs without any cell membrane constraints and avoiding cell toxicity. This growing field offers the opportunity of producing valuable compounds using sustainable biosynthesis. In the ethanol industry, commonly accepted thresholds required for industrial production are yields of 90%, productivity of 1 g/L/h, and titers of 40 g/L.^202^ Some of the discussed network final product titers are 12.5 ± 0.3, 14.9 ± 0.6, and 15.9 ± 0.4 g/L and yields of 88.3 ± 4.6%, 103.9 ± 8.1%, and 94.5 ± 4.7% for limonene, pinene, and sabinene, respectively.^200^ These final product values are close to the threshold values used in the ethanol industry, and it gives high hopes that cell-free biosynthesis will be applied for industrial purposes.

### 6.2 CO_2_ as Substrate

**Figure 9:**
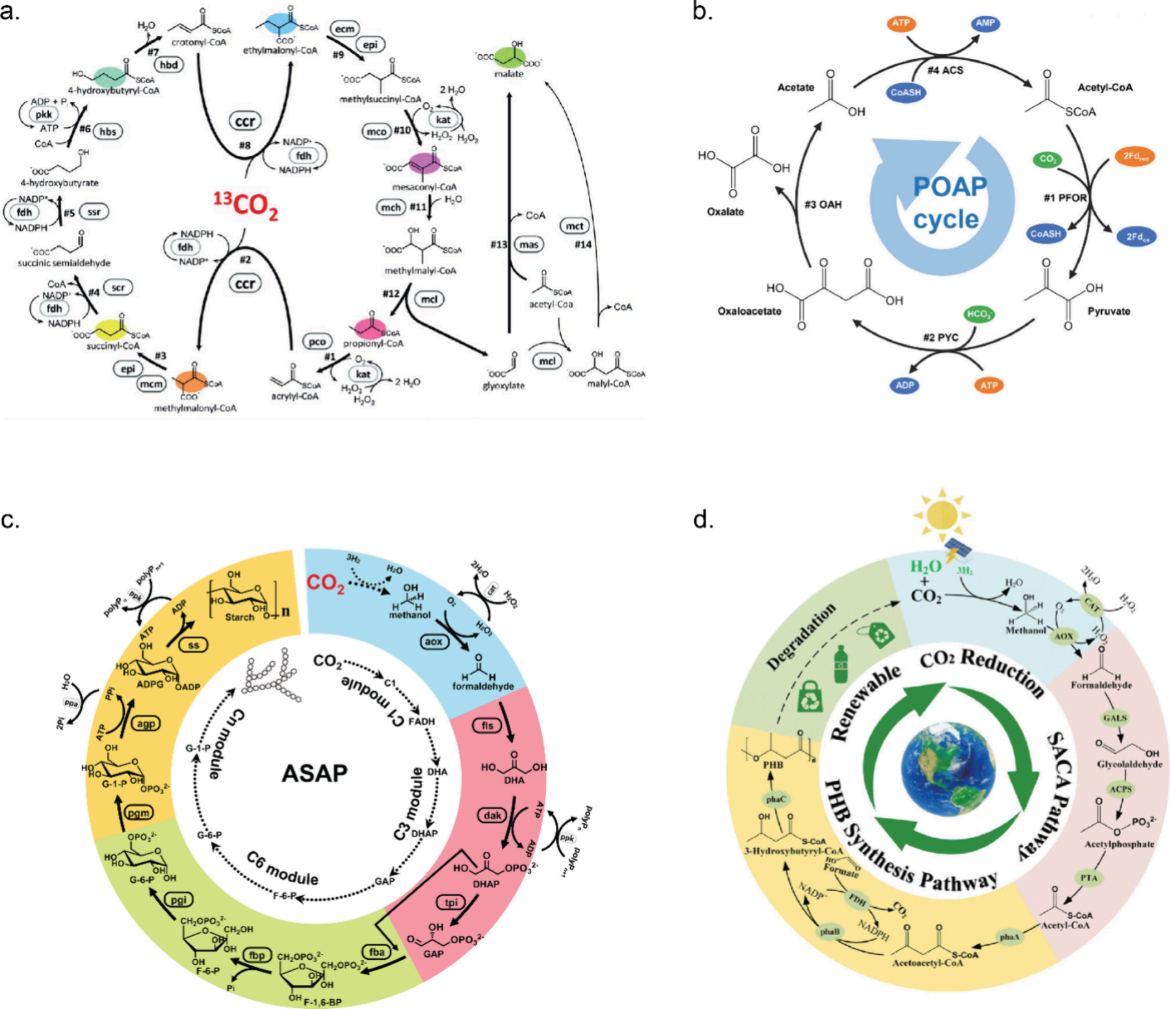
Synthetic CO_2_ fixation pathways. (a) CETCH cycle converting CO_2_ to malate. Adapted with permission from ref (214). Copyright 2016 The American Association for the Advancement of Science. (b) POAP cycle converting CO_2_ to oxalate. Adapted with permission from ref (219). Copyright 2022 American Chemical Society. (c) Chemoenzymatic ASAS pathway converting CO_2_ to starch. Adapted with permission from ref (221). Copyright 2021 The American Association for the Advancement of Science. (d) Chemoenzymatic pathway converting CO_2_ to bioplastic polyhydroxybutyrate (PHB). Adapted with permission from ref (223). Copyright 2023 Royal Society of Chemistry.

In addition to glucose, CO_2_ is also a versatile starting material for the sustainable synthesis of valuable compounds. Many inorganic and metallic catalysts, like SnO_2_, TiO_2_, Cu, and transition metal complexes, have been successfully developed as catalysts to reduce CO_2_.^209^ These inorganic catalysts in general show low selectivity; hence, the products are mainly limited to C1 and C2 compounds, including methanol and ethanol. Nature has evolved several metabolic networks to reduce CO_2_ into higher carbon compounds. However, many of the key carboxylase enzymes that couple CO_2_ to organic substrates have low efficiency.^210−212^ A new-to-nature carboxylase, glycolyl-CoA carboxylase, was recently developed and used in the successful *in vitro* implementation of the previously hypothesized tartronyl-CoA (TaCo) pathway, which had been proposed as a direct route for the assimilation of glycolate into central carbon metabolism and expected to outperform all naturally evolved glycolate assimilation routes.^213^ In contrast, *in vitro* construction of artificial CO_2_ fixation ERNs by freely combining different enzymes from various biological sources is another promising approach. The Erb group assembled the so-called crotonyl–coenzyme A (CoA)/ethylmalonyl-CoA/hydroxybutyryl-CoA (CETCH) cycle with 17 enzymes from nine different organisms to convert CO_2_ into malate at a rate of 5 nmol of CO_2_ per minute per milligram of protein, providing a seventh synthetic alternative to the six naturally evolved CO_2_ fixation pathways (Figure 9a).^214^ This is comparable to the CO_2_ fixation rate (1–3 nmol of CO_2_ per minute per milligram of protein) of the Calvin–Benson–Bassham (CBB) cycle, which fixes more than 90% of carbon in nature.^215^ Moreover, this CETCH cycle could be solar powered through the incorporation of chloroplast extracts.^216^ Subsequently, the output glyoxylate from the CETCH cycle can be applied to produce acetyl-CoA, the central precursor for many natural compounds. Hence, a series of value-added compounds, including monoterpenes, sesquiterpenes, polyketides, and 6-deoxyerythronolide B, has been successfully produced from CO_2_ in one pot based on the CETCH cycle.^217,218^ In addition, a small ERN with four enzymes (pyruvate carboxylase–oxaloacetate acetylhydrolase–acetate-CoA ligase–pyruvate ferredoxin oxidoreductase), named the POAP cycle, was constructed to couple two CO_2_ into one oxalate at the expense of two ATP and one NAD(P)H, reaching a rate of 8 nmol/min/mg CO_2_-fixing enzymes (Figure 9b).^219^ For a more detailed overview, the enzymatic conversion of CO_2_ has been discussed in a recent review on the topic.^220^

### 6.3 Other Substrates

In addition to using sugar and CO_2_ as substrates, progress has been made on fabricating ERNs to upgrade sustainable C1 and C2 stocks, like methanol, ethanol, and acetate, to compounds with longer chains and hence higher value. Cai et al. designed and constructed an 11-enzyme ERN to convert C1 methanol to polymeric starch (Figure 9c).^221^ Zhou et al. designed several ERNs to convert methanol to ethylene glycol, glycolic acid, and d-erythrose.^222^ In addition, the Bioplastic PHB has been produced from methanol and acetate (Figure 9d).^223,224^ Furthermore, Liu et al. managed to use ethanol to produce acetyl-CoA and regenerate ATP using the isoprenol production pathway.^225^

## 7 Future Development

The ERNs discussed so far exhibit a diverse range of functionalities and emergent properties. Yet, they remain relatively small based on several reactions occurring at once. However, as the sections on control over enzyme reactivity and the construction of elaborate reaction cascades for the synthesis of complex organic molecules have demonstrated, the field is now in a strong position to develop ERNs with much more advanced functionalities. In this section, we will discuss two such possible functions (without the ambition of providing complete overviews of those fields): synthetic cells and molecular computers.

### 7.1 Toward Building a Synthetic Cell from the Bottom Up

**Figure 10:**
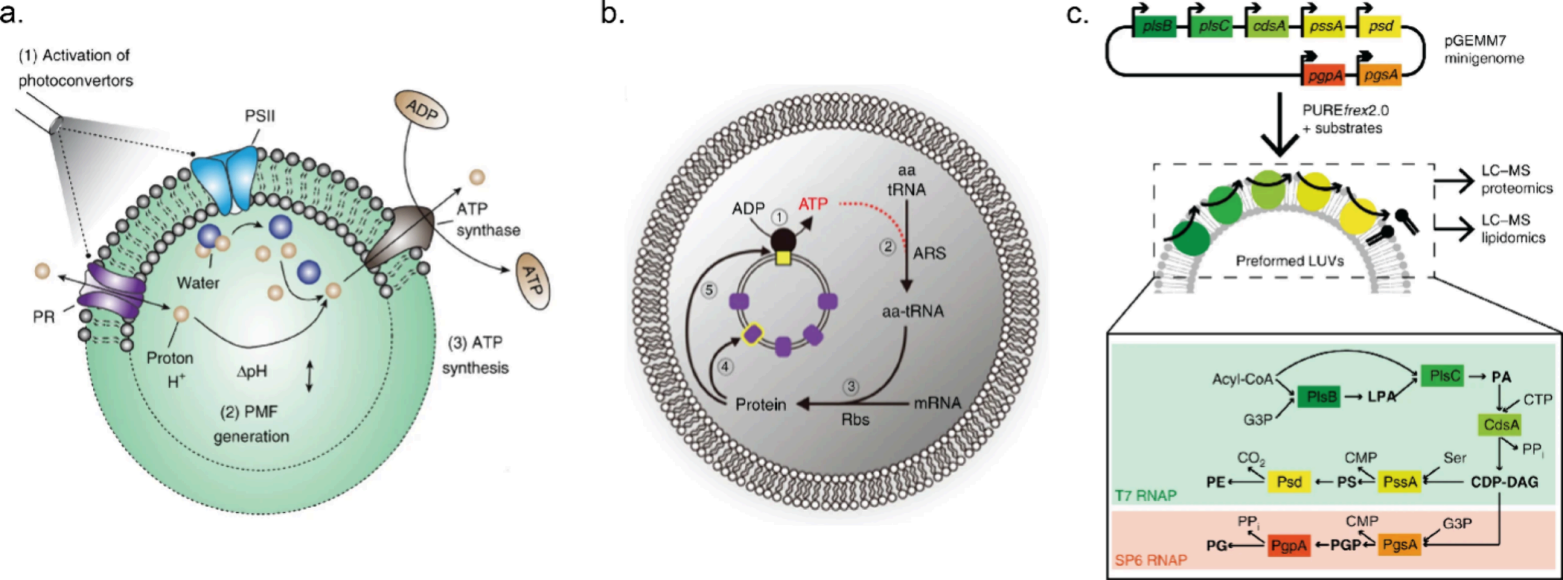
(a) Schematic of the artificial organelle harvesting light to produce ATP and to drive endergonic reactions. Adapted with permission from ref (227). Copyright 2018 Springer Nature. (b) Schematic of self-constituting protein synthesis in artificial photosynthetic cells. Adapted with permission from ref (228). Copyright 2019 licensed under CC 4.0 Springer Nature. (c) Schematic of the production of phospholipids PE and PG by de novo-synthesized enzymes. Adapted with permission from ref (230). Copyright 2020 licensed under CC 4.0 Springer Nature.

Building a synthetic cell from the bottom up could address the fundamental questions on how life emerged and evolved from nonliving components and at the same time generate a wide range of applications. Any functioning synthetic cell will inevitably require the construction of (minimal) metabolic ERNs for the continuous supply of building blocks and energy to support other cellular processes and also response to external environmental changes.^226^ ATP fuels many cellular processes and also serves as a building block for gene transcription and translation. Lee et al. successfully designed and built a photosynthetic artificial organelle consisting of an ATP synthase and two photoconverters in a giant vesicle. This synthetic organelle was capable of harvesting light to produce ATP and to drive endergonic reactions, like pyruvate carboxylase-mediated carbon fixation and actin polymerization into filaments (Figure 10a).^227^ Similarly, Berhanu et al. designed a light-harvesting artificial organelle, which was composed of two kinds of membrane proteins—bacteriorhodopsin and F-type ATP synthase. In this work, the organelle, reconstituted inside a GUV together with a cell-free protein synthesis system, generated ATP required in transcription and at the same time powered the synthesis of GTP and translation (Figure 10b).^228^ Additionally, Bailoni et al. reconstituted the l-arginine breakdown pathway of three cytosolic enzymes to phosphorylate ADP into ATP to fuel the sustainable formation of phospholipid headgroups in synthetic cells.^229^ Blanken et al. managed to encode seven phospholipid-producing enzymes in a synthetic minigenome, which were then expressed within cell-like liposomes. This de novo-constructed ERN can make use of fatty acyl coenzyme A and glycerol-3-phosphate as precursors to produce two phospholipids phosphatidylethanolamine (PE) and phosphatidylglycerol (PG) simultaneously (Figure 10c). The balance of PE and PG is transcriptionally regulated by the activity of specific genes together with a metabolic feedback mechanism.^230^ In addition, Partipilo et al. constructed a small ERN with three enzymes in liposomes to control the redox status of NADH and NADPH cofactors.^231^

Living cells maintain physicochemical homeostasis (including pH, ionic strength, osmotic pressure) to enable the internal components to function near their optimum. This homeostasis state was successfully achieved inside a cell-like system by Pols et al., who co-reconstituted the arginine-breakdown pathway and an ionic strength-gated ATP-driven osmolyte transporter inside vesicles. The cell-like vesicles shrank under increased medium osmolality, causing the internal ionic strength and pH to increase, leading to the inactivation of enzymes. Once the internal ionic strength reached a critical value, the transporter was activated and glycine betaine was pumped in with the accompaniment of the influx of water into the vesicles. Hence, the volume of vesicles increased, and the internal ionic strength and pH decreased. As a result, homeostasis of the ATP/ADP ratio, internal ionic strength, and pH was achieved.^232^ All of these examples clearly demonstrate how enzymatic modules are crucial for mimicking complex metabolic functions of living systems in the field of designing synthetic cells.

### 7.2 Information Processing and Computation

**Figure 11:**
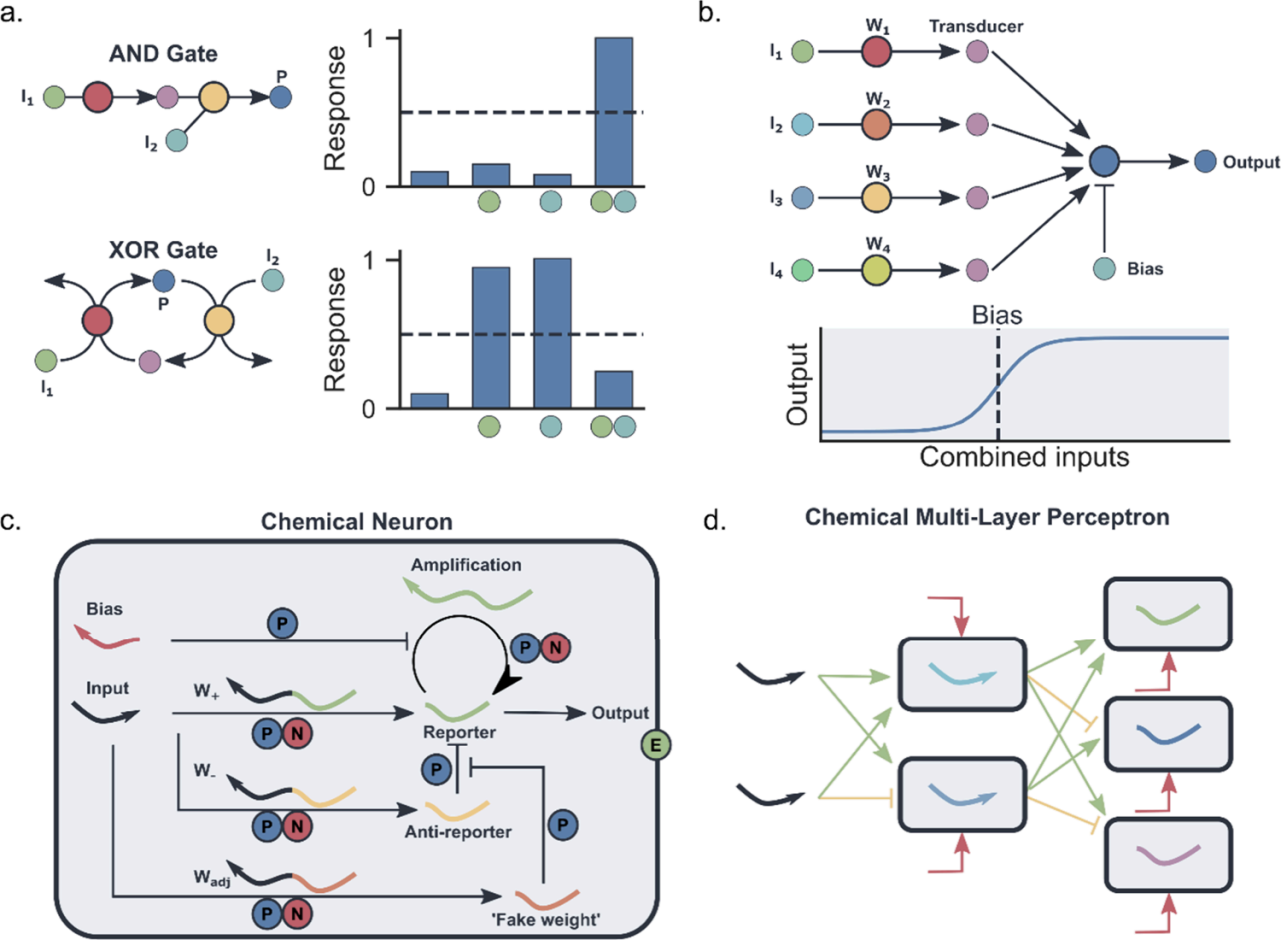
(a) Schematic overview of commonly used ERN designs for two Boolean logic gates (AND and XOR). The computational output is based on an arbitrary threshold for the concentration of a product molecule. (b) A design for an ERN resulting in a multi-input perceptron, investigated in Pandi et al.^238^ The design uses so-called transduction reactions to convert a range of different inputs into the same “transducer” substrate. Weights can be set by modifying enzyme concentrations. The final addition results in a roughly sigmoidal response. (c) Design for a chemical neuron, as investigated by Okumura et al.^239^ A single neuron is constructed from specific DNA templates (curved lines) enabled by components of the polymerase–exonuclease–nickase (PEN) toolbox (shaded circle). (d) Design for a chemical multilayer perceptron. Individual neurons can be combined by replacing fluorescence-generating reporter strands by input strands for other neurons.

An emerging direction for the research on and application of ERNs is special-purpose information processing and computation. So far, the development of Boolean gates and logic circuits has seen a long tradition in enzymatic network research and has been extensively reviewed several times (examples are shown in Figure 11a).^233,234^ However, the development of systems based on digital paradigms has been slowing down, as the principles of Boolean logic and von Neumann computer architectures are challenging to adapt in biochemical systems, which are intrinsically of a nonbinary and dynamic nature. Instead, the potential application of analog and neuromorphic computation principles in biochemistry is increasingly appreciated.^235^ While these developments are still in their infancy, here we focus on recent progress in this direction.

Rather than recreating digital infrastructure directly, recent work on small ERNs has aimed to establish modules and motifs capable of analogue computation. One example of this shows the development of small ERNs that are capable of arithmetic operations.^236^ The key insight here is that specific enzymatic interactions, such as cascading pathways, inhibitors, and parallel product conversion, can be interpreted as arithmetic functions if operated in the right parameter regime. Similarly, specific forms of substrate competition between different enzymes can lead to nonlinear Boolean logic as the theoretical study by Genot and co-workers shows.^237^ This work essentially presents network motifs that act like a nonlinear and nonadditive switch of the starting substrates. This type of research showcases the intrinsic computational power of ERNs, circumventing the requirements necessary for developing explicit digital circuitry.

Recently, this approach has been expanded to enzymatic systems capable of more generalized types of computation. Inspired by concepts from machine learning and neuromorphic computation, Pandi et al. created *in vitro* metabolic perceptrons.^238^ They employed computational retrosynthesis tools to design enzymatic models that can act as transducers (converting different incoming metabolite signals into one substrate) and nonlinear actuators (to achieve sigmoidal responses) and when combined together can act as analogue adders (to perform a weighted sum of incoming signals, e.g., a perceptron). A schematic overview of this is shown in Figure 11b. These perceptrons are essentially based on ultrasensitivity network motifs to create nonlinear weighted adders (sigmoidal sums) of multiple metabolites but do so in a generalizable and extensible manner. Modifying the transducer reactions changes how the incoming signals are weighted, allowing the perceptron to be controlled and trained similar to an artificial neural network.

A different approach is investigated in the work of Okumura et al., where neural networks capable of nonlinear classification have been created by assembling various components of the polymerase–exonuclease–nickase (PEN) toolbox, as shown in Figure 11c.^239^ These enzymes, respectively, polymerize, degrade, and cut DNA strands. Neurons are constructed by supplying specific DNA template strands that convert DNA (or RNA) inputs either into a “reporter” DNA strand or into an “antireporter” DNA strand. The reporter strand can be transformed into a fluorescent signal by another DNA template strand. The antireporter strand polymerizes with the reporter strand, inhibiting its output. Thus, modifying the concentration of reporter and antireporter templates impacts fluorescence, respectively, in a positive or negative way, establishing an effective weight on input conversion. These effective weights can be further modified by incorporating so-called “fake” weight templates, which competitively inhibit the antireporter–reporter interaction. The reporter signal is further amplified by an amplification template, resulting in an ultrasensitive (sigmoidal) response. Finally, the location of the ultrasensitive regime can be adjusted by inclusion of a “bias” template, which operates similarly to the antireporter strand but is not input dependent. Polymerase and nickase drive these DNA reactions, while exonuclease can be used to degrade the fluorescent output over time. The interaction of all enzymes and DNA template strands together establishes a perceptron where all weights and biases can be fine tuned by controlling the concentration of the reporter, antireporter, fake, and bias template strands. By exchanging the reporter templates for strands that can serve as input to another perceptron (Figure 11d), a multilayer neural network was created that could accurately classify input concentration regimes. Importantly, the computational power in this work originates from the highly heterogeneous substrates used in the three-enzyme ERN, in contrast to the previous example of a metabolic network where it originates from the design of the ERN itself.

The above examples show the significant progress that has been made in enzymatic information processing by directly exploiting similarities between enzymatic interactions and analogous principles instead of recreating small Boolean operations and assembling those in a circuit. By either rationally fine tuning the parameter regime in small existing ERNs, by computationally designing larger ERNs to convert many different substrates into the same products, or by intelligently choosing which substrates (and specifically which templates) to exploit using just a small ERN, impressive feats of computation can be achieved. However, further developments have been mostly theoretical up until now, including work showcasing different enzymatic network motifs capable of noise filtering,^240^ pattern recognition and pulse counting,^241^ and systems that can convert information into work and vice versa.^242^ It remains to be seen if these proposals can be realized experimentally, but they highlight the potential for ERNs in future information processing tasks.

## 8 Conclusion

We have presented this comprehensive review of the field of *in vitro* enzymatic reaction networks in the hope to inspire a broad group of researchers to participate in expanding the field. Key design principles, basic emergent properties, and the need for an integrated approach comprising experiments, computational approaches, and analytical methods have been highlighted. Several key challenges for future research were identified: there are currently no “blueprints” for the design of ERNs with functions that go beyond “simple” temporal behavior. For example: we know how to create oscillatory networks, but we cannot design networks that design with a specific frequency and amplitude. Most ERNs reported to date have relatively simple topologies. To significantly expand current designs, we need to establish new strategies for the reverse design of functional ERNs and establish high-throughput methods for the rapid evaluation of networks consisting of multiple feedback loops. Instead of current “trial-and-error” experiments to optimize network properties, iterative “design–build–test–learn” cycles need to be established, and we are hopeful that advances in machine learning and artificial intelligence could be leveraged to identify novel network topologies with hitherto “undesignable” properties. We note that many experimental designs (especially if they are aimed at constructing materials with life-like properties) can only be realized within a relatively narrow scope of kinetic values. Simulations can significantly accelerate discoveries of the most relevant experimental regime, but this will need to be coupled to methods to control or expand the kinetic parameters of enzymes. Although current networks are incomparable in complexity to ERNs found in extant life, our overview on possibilities to control enzymatic activity together with the many examples of feedback loops gives us confidence significantly more elaborate reaction networks will be within reach in the next decade. Indeed, the successful attempts to construct complex reaction cascades and cycles to synthesize complex organic molecules are testament to the potential of more complex ERNs.

We foresee many new developments in the construction of life-like materials, where properties such as homeostasis, sensing the environment, and motility are all carried out by coupled enzymatic reaction networks. The design of intelligent materials by incorporating ERNs, which are able to learn from their environment to adopt desired features, could open up innovative avenues in material science. In section 4, we have explored the influence of external stimuli to regulate ERN activity, serving as physical learning rules for enhanced task performance. Interestingly, these materials potentially can exhibit task adaptiveness through training with different stimuli.^243^ Another important direction for designing life-like materials would be to incorporate some type of fuel regeneration and methods to maintain such materials out of equilibrium as many of the properties associated with living systems (motility, homeostasis, regeneration) require dissipative systems and continuous input of energy. In section 5, we have introduced different strategies like energy production, compartmentalization, and communication using ERNs, which facilitate crosstalk between multiscale processes to design truly autonomous systems.^244^ Such materials could then find applications as delivery vehicles in medicine, in soft robotics, or as interfaces between living systems and electronic systems. In section 6, we demonstrated the progress in cell-free biosynthesis, showcasing the *in vitro* design of metabolic modules for unraveling complex metabolic machinery and producing value-added chemicals. Looking ahead, we anticipate the development of ever more complex ERNs for designing novel catalytic networks with promising industrial applications. Significantly, this can augment the design of novel synthetic cells as chemical factories. Finally, enzymes are key in processing information in biology, but the potential of synthetic ERNs to achieve similar capabilities remains largely unrealized.^245^ Building on the multifarious uses of enzymes in complex (and not so complex) reaction networks, there is much potential for designing novel computing paradigms in which enzymes are used to process multimodal and multiplexed information at the molecular level.

## Abbreviations

1,6-DHN: 1,6-dihydroxynaphthalene
α-HL: α-hemolysin
ABTS: 2,2′-azinobis(3-ethylbenzothiazoline-6-sulfonic acid)
AcAc: acetylacetone
ACAT: acetyl-CoA acetyltransferase
acetyl-CoA: acetyl coenzyme A
AchE: acetylcholine esterase
ADH: alcohol dehydrogenase
ADP: adenosine 5^/^-diphosphate
AMG: amyloglucosidase
AMP: adenosine 5′-monophosphate
Ap: aminopeptidase
ATP: adenosine 5′-triphosphate
Cat: catalase
CBGA: cannabigerolic acid
CETCH: crotonyl-coenzyme A (CoA)/ethylmalonyl-CoA/hydroxybutyryl-CoA
CFPS-ME: cell-free protein synthesis-driven metabolic engineering
CFPS: cell-free protein synthesis
CFME: cell-free metabolic engineering
Cg: chymotrypsinogen
CoA: coenzyme A
COx: choline oxidase
Cr: chymotrypsin
CytC: cytochrome *C*
DASAs: donor–acceptor Stenhouse adducts
DNA: deoxyribonucleic acid
DS: dextransulfate
*E. coli*: *Escherichia coli*
Els: elastase
ERNs: enzymatic reaction networks
Est: esterase
FAD: flavin adenine dinucleotide
FADH_2_: flavin adenine dinucleotide (hydroquinone form)
G6PDH: glucose-6-phosphate dehydrogenase
Gap: glyceraldehyde-3-phosphate dehydrogenase
Gnd: 6-phosphogluconate dehydrogenase
GOx: glucose oxidase
GPb: glycogen phosphorylase b
GPP: geranyl-pyrophosphate
GPPS: geranyl diphosphate synthase
GUVs: giant unilamellar vesicles
Hbd2: hydroxybutyryl-CoA dehydrogenase
HMGR: hydroxymethylglutaryl-CoA reductase
HMGS: hydroxymethylglutaryl-CoA synthase
Hbd: hydroxybutyryl-CoA dehydrogenase
HK: hexokinase
HRP: horseradish peroxidase
IDI: isopentenyl pyrophosphate isomerase
LDH: l-lactate dehydrogenase
LO: l-lactate oxidase
LS: limonene synthase
M23: mutant CBGA synthase
mGap: mutant glyceraldehyde-3-phosphate dehydrogenase
MK: mevalonate kinase
MM: Michaelis–Menten
MMP: matrix-metalloproteinases
NAD: nicotinamide adenine dinucleotide
NADP: nicotinamide adenine dinucleotide phosphate
NO: nitric oxide
NOxE: NADH oxidase
NphB: wild-type prenyltransferase
OA: olivetolic acid
PAAM: polyacrylamide
PBG: pentose–bifido–glycolysis
PDDA: poly(diallyldimethylammonium chloride)
PDH: pyruvate dehydrogenase
PE: phosphatidylethanolamine
PEG: polyethylene glycol
PEGMA: poly(ethylene glycol) methacrylate
PEN: polymerase–exonuclease–nickase
PEP: phosphoenolpyruvate
PG: phosphatidylglycerol
PGLA: poly(lactic-*co*-glycolic acid)
PhaC: PHB synthase
PHB: polyhydroxybutyrate
PKA: protein kinase A
PLA2: phospholipase A2
PMD: pyrophosphomevalonate decarboxylase
PMG: phosphoglucomutase
PMK: phosphomevalonate kinase
PO: peroxidase–oxidase
PP1: protein phosphatase-1
STI: soybean trypsin inhibitor
RLuc: renillaluciferase
RNA: ribonucleic acid
SOx: sarcosine oxidase
TaCo: tartronyl-CoA
Tg: trypsinogen
Tr: trypsin
UOx: urate oxidase
Ur: urease
UV: ultraviolet
